# *Staphylococcus aureus* in Inflammation and Pain: Update on Pathologic Mechanisms

**DOI:** 10.3390/pathogens14020185

**Published:** 2025-02-12

**Authors:** Fernanda S. Rasquel-Oliveira, Jhonatan Macedo Ribeiro, Geovana Martelossi-Cebinelli, Fernanda Barbosa Costa, Gerson Nakazato, Rubia Casagrande, Waldiceu A. Verri

**Affiliations:** 1 Laboratory of Pain, Inflammation, Neuropathy, and Cancer, Department of Immunology, Parasitology and General Pathology, Center of Biological Sciences, Londrina State University, Londrina 86057-970, PR, Brazil; fernandarasquel@uel.br (F.S.R.-O.);; 2 Department of Microbiology, Center of Biological Sciences, Londrina State University, Londrina 86057-970, PR, Brazilgnakazato@uel.br (G.N.); 3 Department of Pharmaceutical Sciences, Center of Health Science, Londrina State University, Londrina 86038-440, PR, Brazil

**Keywords:** immunopathogenesis, infections, virulence factors, MRSA

## Abstract

*Staphylococcus aureus (S. aureus*) is a Gram-positive bacterium of significant clinical importance, known for its versatility and ability to cause a wide array of infections, such as osteoarticular, pulmonary, cardiovascular, device-related, and hospital-acquired infections. This review describes the most recent evidence of the pathogenic potential of *S. aureus*, which is commonly part of the human microbiota but can lead to severe infections. The prevalence of pathogenic *S. aureus* in hospital and community settings contributes to substantial morbidity and mortality, particularly in individuals with compromised immune systems. The immunopathogenesis of *S. aureus* infections involves intricate interactions with the host immune and non-immune cells, characterized by various virulence factors that facilitate adherence, invasion, and evasion of the host’s defenses. This review highlights the complexity of *S. aureus* infections, ranging from mild to life-threatening conditions, and underscores the growing public health concern posed by multidrug-resistant strains, including methicillin-resistant *S. aureus* (MRSA). This article aims to provide an updated perspective on *S. aureus*-related infections, highlighting the main diseases linked to this pathogen, how the different cell types, virulence factors, and signaling molecules are involved in the immunopathogenesis, and the future perspectives to overcome the current challenges to treat the affected individuals.

## 1. Introduction

*Staphylococcus aureus* (*S. aureus*) is a Gram-positive bacterium belonging to the *Firmicutes* phylum [1,2] and is recognized as one of the most relevant pathogens in medical practice due to its high versatility and ability to cause a wide variety of infections [3,4,5]. The importance of *S. aureus* as a clinical pathogen is evidenced by its association with hospital and community-acquired infections [1], being responsible for a significant burden of morbidity and mortality worldwide. In particular, this is due to its broad repertoire of virulence factors and forms of infection [6,7,8].

Under normal conditions, *S. aureus* is commonly found in the microbiota of the skin and mucous membranes of humans, with a colonization rate ranging from 20 to 40% in healthy individuals [2,9,10,11]. Although often harmless, *S. aureus* can become pathogenic when the skin and mucosal barriers are compromised (e.g., in cases of deep wounds, chronic skin conditions, or surgical interventions) [12,13].

Furthermore, colonization by *S. aureus* often precedes the development of infections, especially in individuals using invasive medical equipment (e.g., venous catheters) or in individuals with compromised immune systems [2,13,14]. These factors increase vulnerability to infections and the disease’s development by allowing *S. aureus* to invade the underlying tissues and bloodstream [12].

**Figure 1:**
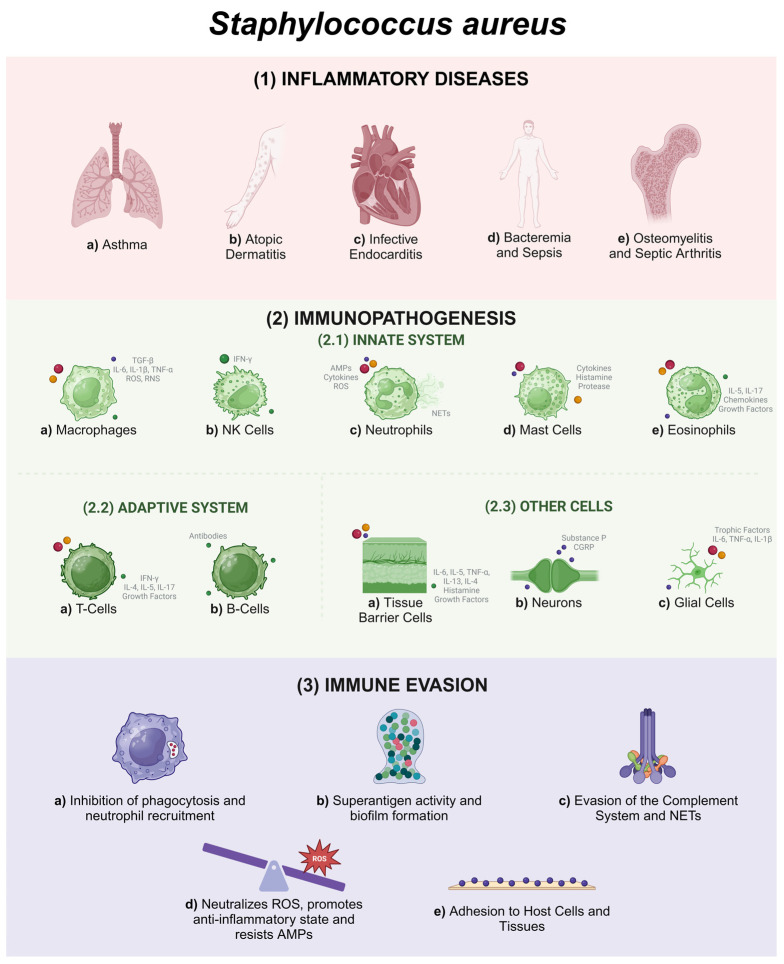
*Staphylococcus aureus*: Inflammatory Diseases, Immunopathogenesis, and Immune Evasion Mechanisms. *Staphylococcus aureus* (*S. aureus*) is an opportunistic pathogen that causes various inflammatory diseases (**1**), including asthma (**1a**), atopic dermatitis (**1b**), infective endocarditis (**1c**), bacteremia and sepsis (**1d**), and osteomyelitis and septic arthritis (**1e**). Following host invasion and bacterial colonization by *S. aureus*, an immune response (**2**) is initiated, involving the innate immune system (**2.1**), the adaptive immune system (**2.2**), and the activation of non-immune host cells (**2.3**). In the innate immune response, cells such as macrophages (**2.1a**), NK cells (**2.1b**), neutrophils (**2.1c**), mast cells (**2.1d**), and eosinophils (**2.1e**) are activated. The adaptive immune response involves the activation of T cells (**2.2a**) and B cells (**2.2b**). Additionally, during immunopathogenesis, other cell types may also become involved, including tissue barrier cells (**2.3a**), neurons (**2.3b**), and glial cells (**2.3c**). All of these contribute to the development of the host’s response against *S. aureus* infection. However, this pathogen has developed mechanisms to evade the host’s immune defenses (**3**), including the inhibition of phagocytosis and neutrophil recruitment (**3a**); superantigen activity and biofilm formation (**3b**); evasion of the complement system and neutrophil extracellular traps (**3c**); neutralization of reactive oxygen species, promotion of an anti-inflammatory state, and resistance to antimicrobial peptides (**3d**); as well as adhesion to host cells and tissues (**3e**). Acronyms: TGF-β—Transforming Growth Factor Beta; IL—Interleukin; TNF-α—Tumor Necrosis Factor Alpha; ROS: Reactive Oxygen Species; RNS—Reactive Nitrogen Species; IFN-γ—Interferon Gamma; AMPs—Antimicrobial Peptides; NETs—Neutrophil Extracellular Traps; CGRP—Calcitonin Gene-Related Peptide.

Infections caused by *S. aureus* range from mild to severe and potentially fatal diseases, such as bacteremia [5], sepsis [5,15], infective endocarditis [16], and osteomyelitis [17,18] (Figure 1). In addition, the significant increase in the prevalence of multidrug-resistant strains of antibiotics, such as methicillin-resistant *S. aureus* (MRSA), has raised major public health concerns [19]. These concerns stem from the difficulty in finding effective treatments against these strains and the increase in associated mortality and morbidity [12,20,21]. Previous studies have reported that mortality from diseases caused by antibiotic-resistant strains has already exceeded 10 million and will possibly surpass cancer death rates by 2050 [19].

The immunopathogenesis of infections and diseases caused by *S. aureus* is complex and involves interactions between the pathogen and the host immune system [12]. These interactions are mediated by innate and adaptive immune cells (e.g., neutrophils, macrophages, and lymphocytes) [15] and by a surprising range of virulence factors (e.g., toxins, enzymes, and cell surface-associated antigens) that are produced by *S. aureus* [20,22] (Figure 1). Successful infection is closely linked to the ability of *S. aureus* to adhere to and invade cells and tissues, as well as evade the host’s defense mechanisms [5,6,13], resulting in exacerbated inflammatory responses, tissue damage, and worsening of infections [5] (Figure 1).

Given this scenario, it is evident that there is a need for research aimed at developing new treatment strategies and deepening the understanding of the mechanisms involved in the immunopathogenesis of *S. aureus* [19,21]. Thus, this review article aims to explore the interactions between *S. aureus* and the diseases generated by it, offering an updated and comprehensive view on the complexity of infections caused by this pathogen and its implications for public health, as well as the future perspectives behind the subject.

## 2. *S. aureus*: Inflammatory Diseases, Current Challenges, and Incidence

**Figure 2:**
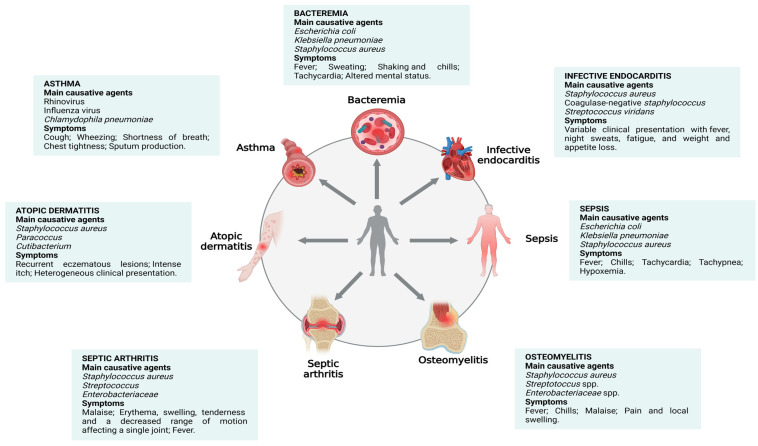
Main microorganisms and symptoms of inflammatory diseases. Several microorganisms are involved in the development and exacerbation of inflammatory diseases, such as asthma, atopic dermatitis, bacteremia, infective endocarditis, osteomyelitis, sepsis, and septic arthritis, resulting in a variety of symptoms [17,29,44,70,107,115].

It is largely known that humans are natural reservoirs of *S. aureus*, which colonizes the skin and mucous membranes [23]. However, *S. aureus* can be an opportunistic pathogen [24], being the cause of bacteremia, infective endocarditis, osteoarticular infection [3], atopic dermatitis (AD) [25], asthma [26,27], and sepsis [28] (Figure 2).

In this section, we will approach the contribution of *S. aureus* in varied disease contexts, which is the base for the relevance of the mechanisms that will be discussed in the following sections.

### 2.1. Asthma

According to the National Institutes of Health Guidelines on Asthma 2007 (The EPR 3 Guidelines on Asthma), asthma is a common and chronic inflammatory disease of the airways. It is characterized by airflow obstruction, recurrent symptoms such as cough, wheezing, shortness of breath, chest tightness, sputum production, bronchial hyperresponsiveness, and underlying inflammation [29]. There are multiple risk factors that lead to the development of asthma, including environmental factors, genetic factors, and physical and psychological conditions [30,31,32,33].

Epidemiological data indicate that this disease affects more than 300 million people worldwide [34]. In 2019, the global prevalence of asthma was 5.42% [35]. However, asthma is also responsible for numerous cases of death, which are influenced by differences in socioeconomic and environmental conditions, mainly in underdeveloped and developing countries, places where it is underdiagnosed and undertreated [36].

However, in recent years, the involvement of *S. aureus* in the development of asthma has gained prominence. One study shows a significant association between nasal colonization of *S. aureus* and asthma prevalence [37]. Another study indicates that environmental exposures to staphylococcal enterotoxins (SE) in houses have the potential to elicit asthma symptoms [38]. Furthermore, it has been demonstrated that specific IgE to *S. aureus* enterotoxins (SE-IgE) is associated with the development of asthma [39]. It was also identified that staphylococcal serine protease–like proteins (Spls) released by *S. aureus* have allergenic properties in humans, as they can induce IgG 4 /IgE antibodies and provoke an immunological response with a type 2 cytokine profile in airway tissues [40]. In addition, it was shown that enterotoxins of *S. aureus*, in addition to acting as conventional antigens leading to a specific IgE response, can act as superantigens leading to polyclonal activation of T cells [41].

Therefore, a possible therapeutic approach for asthma concomitant with *S. aureus* infection is based on anti-IgE, such as omalizumab. This anti-IgE monoclonal antibody has been shown to be effective in decreasing IgE levels, providing improvement in symptoms and quality of life, and reducing the use of concomitant and rescue medications [42].

Although there are several treatments currently available for asthma, the outcomes of interactions of *S. aureus* infection, asthma, and drug sensitivity must be better investigated to provide tailored treatments.

### 2.2. Atopic Dermatitis

Atopic dermatitis (AD), also known as eczema and atopic eczema, is a chronic inflammatory skin disease that is recurrent and multifactorial, affecting thousands of people around the world [43]. The main symptoms associated with this disorder are recurrent eczematous lesions, intense itch, and a diverse clinical presentation, being heterogeneous in terms of severity, clinical signs, and development [44]. The pathophysiology of AD is complex and includes skin barrier dysfunction, dysregulation of the immune system with a more exacerbated Th2 response, and genetic predisposition [44]. Studies have demonstrated significant dysbiosis in the skin microbiome in patients with AD, as shown in Table 1, with greater colonization by *S. aureus*, and this correlated with worsened disease severity [45,46,47,48,49].

Therefore, with changes in the epidermal barrier, infection by opportunistic *S. aureus* can occur, which produces several virulence factors such as toxins, enzymes, and proteins, resulting in inflammation and exacerbation of AD [37]. It has been demonstrated that the δ-toxin is a mast cell degranulation-inducing factor produced by *S. aureus*, while phenol-soluble modulins (PSMs) from *S. aureus* stimulate the release of proinflammatory cytokines, such as IL-18 and IL-1β from keratinocytes, exacerbating skin inflammation [51,52].

Epidemiological data show that AD has a global prevalence of 2.6% and affects approximately 204 million people. Of these, the most affected are children, with a prevalence rate of 4.0% [53]. Therefore, it is more common in childhood, generally appearing in the first five years of life in 85% of cases and disappearing in about 40% of them upon growth [54]. Data also show that colonization by *S. aureus* in AD occurs in more than 90% of cases [54]. However, AD can manifest at any age, and this generates numerous socioeconomic impacts. These impacts can be direct, indirect, or intangible, but they all generate substantial costs, and thus, AD represents a public health problem [55].

### 2.3. Bacteremia

Bacteremia is defined as the presence of pathogenic bacteria in the bloodstream [56]. Examples of bacteria that can cause bacteremia are shown in Table 2.

*S. aureus* stands out as a leading cause of community-acquired and healthcare-associated bacteremia [61]. There are numerous risk factors for *S. aureus* bacteremia, such as age (high rates in the first year of life and in the elderly), underlying medical conditions, ethnicity, intravenous drug abuse, frequent skin and soft tissue infections, and the presence of intravascular catheters, in addition to pulmonary and osteoarticular infections [3,62].

*S. aureus* has several mechanisms to resist and escape the host’s immune response in the bloodstream. The secretion of coagulases is capable of leading to prothrombin activation, and the presence of agglutinins on the surface of *S. aureus* makes them capable of binding to fibrin, forming large bacterial aggregates covered with fibrin. This mechanism allows the bacteria to escape phagocytosis and culminates in the formation of abscess lesions [16]. These lesions eventually rupture, releasing purulent exudate and staphylococci, which disseminate and produce new infectious lesions or infect new hosts [16]. 

Currently, the gold standard for the diagnosis and treatment of bacteremia is to obtain blood cultures as early as possible [63], although there are other less-used molecular-based tests that provide faster results [64,65]. In addition, some principles are also used, including defining if the patient has a complicated or uncomplicated infection, identifying and removing infected foci, and finally, administering appropriate antimicrobial therapy in relation to the causative agent, dose, and duration [3]. For bacteremia caused by methicillin-susceptible *S. aureus* (MSSA), β-lactams are used as treatment, such as isoxazolic penicillins (cloxacillin) and first-generation cephalosporins (cefazolin), due to their faster and more intense bactericidal activity against MSSA, while for bacteremia caused by methicillin-resistant *S. aureus* (MRSA), vancomycin and daptomycin are used, the activity of the latter being more intense and faster [66].

Research shows that the global incidence of *S. aureus* bacteremia is approximately 30 cases per 100,000 people per year [61,67]. Meanwhile, mortality rates have decreased over the years with the introduction of antibiotics, resulting in approximately 2 to 10 deaths annually per 100,000 population [68]. However, data show a high mortality rate related to MRSA bacteremia and infective endocarditis, from 17% to 50% [61]. 

### 2.4. Infective Endocarditis

Infective endocarditis (IE) is an inflammatory disease that results from infection of the endocardium, affecting the inner lining of the heart and heart valves [69]. IE has a variable clinical presentation, which can be acute, subacute, or chronic, as it can be caused by different microorganisms (as shown in Table 3) and reflects underlying cardiac conditions or pre-existing comorbidities [70].

Studies show that the most common causative agent of IE is *S. aureus* [73,74]. The pathophysiology of IE begins with damage to the cardiac endothelium, which leads to exposure of subendothelial cells, resulting in the production of extracellular matrix proteins, tissue factors, and the deposition of fibrin and platelets, forming sterile vegetations [3]. Therefore, bacteria in the bloodstream can adhere to these vegetations, leading to additional deposition of fibrin and bacterial proliferation, causing IE [69]. It has been demonstrated that superantigens secreted by *S. aureus*, in addition to evading the host immune response, are critical virulence factors in IE, as they result in the formation of vegetations through superantigenicity and direct toxic effects on aortic endothelial cells [75].

The global incidence of IE is estimated at 3–7 cases per 100,000 people per year, and the hospital mortality rate ranges from 15 to 30% [76]. Furthermore, the incidence of hospitalizations increased in recent years, which led to an increase in medical and hospital expenses [77]. Therefore, IE is a disease that generates high socioeconomic impact, mainly related to hospitalization and treatment costs.

### 2.5. Osteomyelitis

Osteomyelitis is an inflammatory disease in the bones that is caused by infection [78]. It is characterized by intense inflammation, vascular damage, and localized bone loss [79]. Bone infections may be secondary to a contiguous focus of infection or by direct inoculation (for example, after trauma or surgery), result from vascular insufficiency and infection of surrounding soft tissues with the bone (e.g., diabetic foot), or occur through hematogenous routes (more common in children) [78,80]. Several microorganisms can cause osteomyelitis, as shown in Table 4.

The most common microorganism causing osteomyelitis is *S. aureus* [17]. It is responsible for both acute and chronic osteomyelitis through biofilm formation, antimicrobial resistance, and expression of virulence factors [80]. Several studies have reported the role of *S. aureus* in osteomyelitis [83]. *S. aureus* is capable of mediating bone destruction, either by promoting the activation of osteoclasts, leading to an increase in the release of pro-inflammatory cytokines and activation of the transcription factor NF-ĸB, or by inducing osteoblast apoptosis [84,85].

Previous data show that the annual incidence of osteomyelitis was 21.8 cases per 100,000 persons, with the incidence being higher for men and increasing with age [86]. More recent data show that osteomyelitis significantly affects children aged 10 to 15 years with an incidence of 15.3/100,000 [87].

### 2.6. Sepsis

The Third International Consensus Definitions for Sepsis and Septic Shock (Sepsis-3) defined sepsis as “life-threatening organ dysfunction caused by a dysregulated host response to infection”. Therefore, sepsis is characterized by both physiological, pathological, and biochemical abnormalities resulting from an infection [88]. Several microorganisms can cause sepsis, as can be seen in Table 2, but *S. aureus* stands out.

Several studies have demonstrated the incidence of sepsis caused by *S. aureus*. A study involving 74 patients with SAB and IE in Finland showed that 50% had severe sepsis [89]. Meanwhile, another study with 373 patients with *S. aureus* bloodstream infection in Norway showed that 57.4% of patients had sepsis without organ failure and 29.8% had severe sepsis [90]. Other studies show that *S. aureus* is able to up-regulate virulence factors under stress and thus persist in the bloodstream, and that high-level superantigens (SAgs) are critical in lethal sepsis [91]. Because of this, the presence of *S. aureus* in the bloodstream can lead to sepsis [92].

The pathophysiology of *S. aureus* sepsis involves several factors and is mediated by inflammation and immunosuppression, which may occur concurrently [93]. Initially, the host response to pathogens occurs through the production and release of proinflammatory and immunomodulating cytokines [94]. Monocytes/macrophages are cells of the monocytic lineage that are critical in bacterial sepsis, as they are the main source of inflammatory cytokines that are responsible for septic shock, such as tumor necrosis factor alpha (TNF-α), IL-1β, and IL-6 [94]. However, disorders in innate and acquired immunity can occur, leading to immunosuppression, with the release of anti-inflammatory cytokines, death of immune cells, T-cell exhaustion, and expansion of regulatory immune cells [93]. Thus, this uncontrolled inflammatory response in sepsis can lead to disseminated intravascular coagulation, organ failure, and tissue damage, leading to septic shock, rendering it as one of the causes of high mortality [93].

Therefore, because sepsis is complex, more recent recommendations and guidelines for the treatment of sepsis have been published through the Surviving Sepsis Campaign (SSC) [95]. They include screening and early treatment, diagnosis of infection and treatment, hemodynamic management, ventilation, and additional therapies [95]. Some of these treatments include hemodynamic resuscitation with the use of crystalloids, antimicrobial therapy, and corticosteroids, considering the physiological parameters of each patient [96].

With improvements in treatments over the last two decades, mortality from sepsis has reduced [93]. However, there are still significant mortality rates, as a study showed that in 2017, 48.9 million cases of sepsis were recorded worldwide and that 11 million sepsis-related deaths were related, a fact that is associated with diverse underlying conditions and varying immune responses among patients [97,98]. A recent study showed that the global annual sepsis incidence was 276–678/100,000 people, while lethality ranged from 22.5 to 26.7% [99]. Furthermore, a study in Japan showed that medical costs to treat sepsis amounted to US $4.38 billion in 2017 [100]. Therefore, sepsis still causes many deaths, in addition to a high cost related to treatment, which represents a major health and socioeconomic problem for the world [101].

### 2.7. Septic Arthritis

Septic arthritis is an inflammatory disease caused by an infectious agent, which mainly affects large joints, such as the knee or hip [102]. Among the causative agents, as shown in Table 5, the most common is *S. aureus*, which presents a pronounced role in patients with rheumatoid arthritis or diabetes [103].

Generally, septic arthritis is considered a secondary infection due to bacterial dissemination from the bloodstream to surrounding tissues, significantly increasing morbidity and mortality [106]. However, in addition to the hematogenous route, there are other routes through which pathogens can enter the synovial membrane, infecting the joint, which are infected contiguous foci, neighboring soft-tissue sepsis, or direct inoculation due to trauma or an iatrogenic event such as intra-articular administration of medicine [107].

Therefore, once inside the synovial space, bacteria will adhere to the synovial cells, multiply rapidly, and bacterial products and toxins will initiate the inflammatory response [108]. There is the release of IL-1β and IL-6 into the synovial fluid, which leads to the release of acute-phase proteins from the liver, promoting opsonization and activation of the complement system [103]. There is also an influx of immune cells that leads to the release of TNF-α, IL-8, and granulocyte-macrophage colony-stimulating factor (GM-CSF) [103]. TNF-α, IL-1β, and IL-6 have been indicated as being the main cytokines that lead to severe inflammation that precedes cartilage and bone destruction in septic arthritis [102]. Other cytokines such as IL-33 limit the immune response in septic arthritis; thus, inhibiting its signaling through ST2 receptors enhances Th1 lymphocytes and IFNγ production, resulting in optimized nitric oxide production and bacterial killing [109].

Furthermore, Toll-like receptors (TLRs) will act by recognizing bacterial pathogen-associated molecular pattern molecules (PAMPs) and activating the transcription factor kappa B (NF-κB). The continuous phagocytosis of bacteria will culminate in a greater release of pro-inflammatory cytokines in an attempt to eliminate the infection [108,110]. If the infection is not eliminated, there is a large influx of T-cells and macrophages, causing a thickening of the synovial membrane [108]. In addition, due to high levels of cytokines, matrix metalloproteinases (MMPs) will be released from the host’s cells, contributing to the progressive destruction of joints in septic arthritis mediated by *S. aureus* [111].

Therefore, the standard treatment for septic arthritis consists of irrigation and debridement of the affected joint through arthroscopy, in addition to the administration of intravenous antibiotics [105]. Studies in animal models have demonstrated that combined antibiotic therapy with nonsteroidal anti-inflammatory drugs or corticosteroids is capable of reducing cartilage damage in staphylococcal septic arthritis and decreasing the prevalence, severity, and mortality of the disease, respectively [112,113]. However, in humans, there are still more clinical trials needed to provide other treatment alternatives, as this standard treatment has been used for more than two decades [28].

Recent epidemiological data indicate that the annual incidence of septic arthritis varies from 1 to 35 cases/100,000 people [105]. People over 80 years and young children are the most affected [107]. However, a study showed that the incidence may vary according to ethnicity and increase with socioeconomic deprivation and age [114]. Therefore, being a rapidly progressing disease that causes significant joint damage, people with low socioeconomic conditions, children, and the elderly are directly impacted, representing a clinical emergency.

In summary, Section 2 brought an overview of diseases with the involvement of *S. aureus* in their etiology (Figure 2).

Hence, as *S. aureus* is associated with several diseases with significant inflammatory components that commonly dictate severity, we discuss below the immunopathogenesis and how virulence factors and other evasion mechanisms put staphylococci infections in the spotlight.

## 3. Immunopathogenesis: Host Reactivity During *S. aureus* Infections

Upon host invasion and bacterial colonization by *S. aureus*, an intense immune response is established, and several pro-inflammatory mediators are produced by immune cells such as neutrophils, macrophages, and B and T lymphocytes, as well as by non-immune cells. Besides cytokines and chemokines as potent leukocyte chemoattractants, it was previously described that bacterial products can directly impact neutrophil recruitment [116,117]. The interplay between pathogen-derived virulence factors and host immune reaction directly dictates the severity and/or resolution of the infection. Pre-clinical septic arthritis models show that chronic disease leads to histopathological changes that include local edema, pannus formation, synovitis, cartilage destruction, and bone loss [109,118], and a higher number of complications are seen among immunosuppressed sepsis patients [119].

This section was divided into three main topics: innate immune response, adaptive immune response, and the role of non-immune cells contributing to response against *S. aureus* infection.

### 3.1. Innate Immune Response

As a first line of defense, the innate immune response produces mediators to recruit more cells to the infection foci; at the same time, it induces microbial killing by triggering immune cell and complement system activation. When and where microorganisms invade, various cell types such as epithelial cells sense and recognize *S. aureus* components, including lipoteichoic acid (LTA), phenol-soluble modulins, lipoproteins, protein A, and peptidoglycans (PGN), and start an immune response [94]. This recognition occurs via pattern recognition receptors (PRRs), (e.g., TLRs). Following PRR activation, cytokines, chemokines, and growth factors, for example, IL-6, chemokine (C-X-C motif) ligand 8 (CXCL8), monocyte chemotactic protein-1 (MCP-1), granulocyte colony-stimulating factor (G-CSF), and granulocyte-macrophage colony-stimulating factor (GM-CSF), are released to guide the recruitment and sustain leukocyte survival in the infection site [120,121,122,123]. In this section, we discuss the main cell types participating in the innate immune response against *S. aureus*.

#### 3.1.1. Macrophages

As professional phagocytes, macrophages have essential roles during host response against *S. aureus*. Their activities and signaling can be beneficial or detrimental depending on the context. Tissue-resident macrophages originate from prenatal precursors and are immune sentinels transcriptionally programmed for rapid pathogen recognition and further immune system activation [124]. Furthermore, macrophages can originate from circulating monocytes that are recruited from the bloodstream to the site of the inflammation for effective infection control but can also contribute to the pathogenesis of several inflammatory diseases [125]. Monocytes comprise around 10% of leukocytes in humans, while tissue-resident macrophages are about 15% of total leukocytes (in quiescent conditions) [126,127]. The importance of macrophage activity during *S. aureus* infections is highlighted by studies in which depletion of this cell type has increased bacterial burden and mortality in sepsis [128] and airway infections [129,130,131].

Macrophage chemotaxis is mediated by molecules produced by both host cells and bacteria. The chemokines CCL9 [132] and CCL2/MCP-1 [123,133], the bioactive lipid leukotriene B4 (LTB4) [134], and complement cascade activation [135,136] promote phagocyte infiltration during *S. aureus* infection. Formylated peptides (fMLP) produced by *S. aureus* account for monocyte and neutrophil chemoattraction as well since these phagocytes express formyl-peptide receptors [137,138].

Upon bacterial encounter, macrophages get activated and are polarized to express genes that will favor leukocyte survival and microbial clearance [139]. The nomenclature M1 and M2 macrophage polarization are commonly used to refer to pro-inflammatory and anti-inflammatory phenotypes, respectively. However, M1 and M2 terminologies seem to be an oversimplification—due to the great plasticity of those cells, macrophages are able to express a variety of phenotypes [140]—therefore, this article will refer to inflammatory and anti-inflammatory macrophages for better understanding.

Inflammatory macrophages, as expected, elicit a pro-inflammatory response that enhances bacterial sensing, phagocytosis, ROS and NO generation, and the release of inflammatory cytokines and can express class II major histocompatibility complex molecules (MHC-II) [141]. *S. aureus* peptidoglycan and lipoproteins are recognized by TLR2, leading to downstream MyD88 activation and NF-κB signaling [142,143], a pathway known to elicit pro-inflammatory cytokine production. Inhibition of TLR2 or MyD88 activity significantly impacts cytokine release [144,145], and TLR2 deficiency results in susceptibility to *S. aureus* infection [146]. Furthermore, previous reports state that NF-κB activation is required for phagocytosis by macrophages [147]. This macrophage polarization towards an inflammatory profile via TLR2 is critically dependent on forkhead box O1 (FoxO1) overexpression [148]. In addition to TLR2, TLR4 can also be activated by *S. aureus*: the bacterial component iron-regulated surface determinant protein B (IsdB) promotes IL-6 and IL-1β production by monocytes via the TLR4-MyD88-NF-κB pathway, which also requires activation of the NLRP3 inflammasome [149].

In contrast, anti-inflammatory macrophages have limited phagocytic activity and are associated with chronic inflammation in *S. aureus*-colonized rhinosinusitis patients [150]. In fact, in some *S. aureus* infections, such as the ones affecting catheters or prosthetic joints, bacteria induce biofilm formation to avoid phagocytosis, attenuating traditional host pro-inflammatory response and further stimulating the anti-inflammatory phenotype in monocytes [151,152]. It was previously described that biofilms promote immune response evasion by different mechanisms: responding to host-derived IL-1β, facilitating microbial growth, diminishing pro-inflammatory cytokine expression, and increasing anti-inflammatory macrophages [153,154]. Reprogramming the biofilm-associated monocytes from an anti-inflammatory to an inflammatory profile aids in biofilm clearance, suggesting an interesting therapeutic approach [155]. Moreover, the neuropeptide CGRP seems to promote macrophage polarization towards an anti-inflammatory phenotype during skin infections [156], when *S. aureus* virulence factors (e.g., formyl peptides or HLα, a pore-forming toxin) activate TRPV1^+^ neurons [157].

As in regard to mechanisms employed by macrophages to kill *S. aureus*, phagocytosis is accompanied by several processes to degrade the microorganism: acidification of the phagolysosome, ROS and reactive nitrogen species (RNS) generation, antimicrobial peptides and lysozyme action, and macrophage extracellular traps (mETs) (for more detailed reviews, see [139,158]. The acidification of bacteria-containing phagolysosomes is made by ATPase, an H^+^ pump that expels hydron (H^+^) from the cytosol into the vesicle, lowering the pH to 5.0 or less [159] and creating a hostile environment for bacterial growth [160] and favoring the activity of cathepsins, enzymes with optimal efficacy at lower pH [161]. ROS production depends on NADPH oxidase (NOX) assembly, an enzyme that catalyzes superoxide production and noxious ROS that damage DNA, proteins, and lipids [162], being an important mechanism of action against *S. aureus* [163]. Patients with mutations in one of the subunits of NOX2 are susceptible to *S. aureus* infection [164]. Production of RNS, in turn, is catalyzed by inducible nitric oxide synthase (iNOS) [165], which also contributes to *S. aureus* killing [109]. An inflammatory stimulus (e.g., interferon-gamma [IFN-γ]) is necessary for iNOS expression induction [166]. Antimicrobial peptides and enzymes are additional tools of macrophage-killing machinery. Some examples are cathepsins, proteases known to promote proteolytic attack and influence the production of pro-inflammatory cytokines [167], and lysozymes, able to interfere with peptidoglycan integrity and cause bacterial lysis [168], despite the fact that *S. aureus* seems to be highly resistant to lysozyme activity [169,170]. In addition to neutrophils (discussed in more detail in Section 3.1.3), extracellular traps can be released by other cell types, including macrophages—when they are called mETs—which facilitate bacterial immobilization and killing [171,172,173].

As discussed in Section 2.7, septic arthritis is a debilitating disease characterized by colonization of articular space, and *S. aureus* is the most commonly identified organism in the synovial fluid of these patients [174]. Synovial cavity macrophages, after phagocytosis, act as antigen-presenting cells (APCs) to activate the adaptive immune system [139]. During septic arthritis pathogenesis, macrophages secrete transforming growth factor-beta (TGF-β), IL-6, TNF-α, and IL-1β [175], cytokines with significant roles in NF-κB activation and osteoclast differentiation, perpetuating the inflammatory milieu and bone resorption [176,177]. Ghosh and colleagues [178] described that neutralization of TGF-β and IL-6 ameliorated septic arthritis by modulating tissue-resident macrophages; RANKL/OPG interaction (preventing bone loss); and enhancing the antioxidant activity of superoxide dismutase and catalase [178]. Similarly, by blocking TLR2 activation and consequent TNF-α and IL-1β production, disease severity was mitigated [179]. Moreover, after depleting monocytes with etoposide, lower levels of TNF-α, IL-1β, O_2_^−^, H_2_O_2_, NO, metalloproteinase-2 (MMP-2), RANKL, and osteoprotegerin were detected, attenuating *S. aureus*-induced tissue destruction during septic arthritis in mice [180].

Lipoproteins are components of the *S. aureus* cell wall known for inducing a strong macrophage response, and variants lacking lipoproteins escape immune recognition [181]. As PAMPs, lipoproteins are recognized by macrophages and promote major NO production [182], TNF release [183], and bone resorption due to their lipid-moiety effect through monocytes/macrophages [184], and by shifting cellular metabolism to glycolysis, as lactate accumulation leads to bone erosion [185]. One study conducted by Mohammad and colleagues [183] showed that, in a murine model of septic arthritis, intra-articular injection of lipoproteins resulted in chronic inflammation and joint tissue degradation, while *S. aureus* mutant lacking lipoprotein diacylglyceryl transferase resulted in a more severe outcome with uncontrolled bacterial burden, suggesting the importance of lipoproteins for proper recognition by leukocytes. Co-injection of lipoproteins and viable *S. aureus* boosted neutrophil and macrophage activation, abrogating the severity of the disease, therefore implying an interesting action of lipoproteins as adjuvants of host defenses [183].

In conclusion, macrophages are professional phagocytes chemoattracted to the infection site by molecules produced both by other immune cells and by bacteria and are further activated and polarized to favor leukocyte survival and enhance microbial killing activity. Macrophage machinery includes phagolysosome formation, antimicrobial peptide production, and release of mETs. During septic arthritis pathogenesis, macrophages exert an important role.

#### 3.1.2. Natural Killers (NKs)

Natural killers (NKs) are a subset of innate lymphoid cells (ILCs) important during the innate immune response against tissue injury, microbes, tumors, and the initial phase of inflammation [186,187,188]. During bacterial infections, NKs recognize microorganisms via TLRs and produce IFN-γ to activate macrophages, which in turn produce cytokines and ROS for a robust antimicrobial response [189]. Interestingly, at the same time, NK-derived IFN-γ induces macrophage priming. NKs activation by enterotoxins derived from *S. aureus* is thoroughly dependent on the presence of both monocytes and αβ T cells [190].

The relevance of NKs during *S. aureus* host response is highlighted by studies that demonstrate their protective role during *S. aureus*-induced arthritis [191] and that NK-deficient mice are susceptible to pulmonary staphylococcal infection despite a great influx of macrophages and neutrophils. This process seems to be dependent on NK-derived IL-15 [192]. In addition, type II natural killer T cells are crucial for lowering bacterial burden prior to conventional T cell activation, exerting effector functions against *S. aureus* [193]. Furthermore, after a skin infection, circulating NKs turn into long-lived tissue-resident NKs to respond to secondary infections more quickly, acting as memory-like NK cells [194].

On the other hand, some studies point to the controversial aspect of IFN-γ: while boosting immune response, especially by activating macrophages for phagocytosis, higher survival rates were observed when neutralizing [195] or using IFN-γ-deficient mice [196] infected with *S. aureus*. Bacterial β-hemolysin induces IFN-γ in human CD56^bright^ NK cells, contributing to infection pathogenesis [197]. This dual role may be disease-dependent and multifactorial [198,199].

Therefore, NKs are important players during *S. aureus* infections as they recognize bacteria and activate other cells, such as macrophages via IFN-γ; despite other leukocytes’ presence, animals that lack NKs show defense impairment, and also this cell type can act as memory-like cells. However, NK-derived IFN-γ can have ambiguous roles and may be detrimental by over-boosting inflammation, leading to poor survival rates.

#### 3.1.3. Neutrophils

Human neutrophils, or polymorphonuclear (PMN) leukocytes, comprise 50–70% of the total circulating leukocytes [200] and are professional phagocytes that possess important microbicidal machinery [201]. Proper regulation of the recruitment and activation of these cells is essential because immune suppression can lead to impaired microbial clearance and higher mortality [202,203,204,205], but excessive and prolonged neutrophil infiltration can lead to tissue destruction and organ dysfunction [206].

Upon *S. aureus* invasion, signaling molecules that function as chemoattractants are released by tissue-resident cells, neurons, and bacteria, resulting in the recruitment of inflammatory cells to the site of the infection. *S. aureus*-derived chemotactic molecules, such as LTA and polysaccharides, induce IL-8 production, which promotes transmigration of neutrophils [207,208,209]. In a mouse model of osteomyelitis, *S. aureus* elevated the expression of neutrophil chemoattractants CXCL1, CXCL2, CXCL3, CXCL5, CC chemokine ligand 3 (CCL3), and CCL7 [210]. Evidence suggests that the pro-inflammatory cytokine TNF-α promotes neutrophil recruitment via the intrinsic TNF receptor TNFR1, while TNFR2 directs neutrophil antimicrobial functions against *S. aureus* [211]. Formylated peptides present in *S. aureus* structure also are potent chemoattractants [212]. In fact, combining antibiotics with formylated peptides is an approach proposed by some authors to boost immune response and help combat the infection [213]. In addition, neutrophils are essential for the clearance of *S. aureus* aggregates that form on medical implants [214], and delayed neutrophil recruitment to a contaminated implant site allows *S. aureus* to establish a nascent biofilm and avoid host defenses [215].

Interestingly, neurons are also able to affect neutrophil recruitment (for a more detailed discussion on the role of neurons in *S. aureus* infections, see Section 3.3.2). Transient receptor potential cation channel subfamily V member 1 (TRPV1)+ neurons release calcitonin gene-related peptide (CGRP) during *S. aureus*-induced skin infections, which inhibits local neutrophil infiltration, decreases IL-1β and TNFα while enhancing IL-10 levels, aggravating the infection. In fact, TRPV1 knockout animals displayed better outcomes than their wild-type counterparts during *S. aureus* infection [156]. This study was preceded by the observation that Nav1.8-Cre/Diphtheria Toxin A (DTA) animals (whose Nav1.8^+^ nociceptors were ablated) showed significantly augmented neutrophil recruitment due to the inability to release CGRP, thus demonstrating a detrimental role of nociceptive neurons in *S. aureus* infection [157].

Molecules that serve as a chemoattractant to neutrophils also prime those cells for a more robust response upon bacterial encounter. Molecules produced by the host capable of priming neutrophils include chemokines and cytokines (e.g., TNF-α, IL-1β, IL-6), growth factors (e.g., GM-CSF, G-CSF), and cell-to-cell contact and adhesion, which improve cytokine production and release, phagocytic activity, production of ROS, and degranulation, resulting in more efficient bactericidal activity [216]. *S. aureus* bacterial factors able to prime neutrophils include phenol-soluble modulin peptides [117] and LTA [217,218].

Neutrophils that have been primed and/or recruited by chemoattractants exit the bloodstream to reach the site of infection, where these cells are activated by PAMPs (e.g., LTA from *S. aureus*) binding to their pattern recognition receptors (e.g., TLR-2), recognizing pathogens. Priming and chemotaxis demand a lower stimuli concentration than neutrophil activation. Besides pathogen recognition, activated neutrophils display extended life span, enhanced adhesion, phagocytic activity, and production of cytokines and granules, features that are necessary for a proper antimicrobial response [216,217,219]. Evidence suggests that TLR2 and TLR4 are the main TLRs involved in *S. aureus* immune response [146,220,221,222]. In addition, NOD-like receptors, specifically NOD2, identify muramyl dipeptide derived from *S. aureus*, inducing NF-κB translocation to the nucleus [223,224]. Neutrophils also express complement receptors and mannose-binding lectin receptors. Mannose-binding lectins (MBL) are recognition molecules able to detect molecular patterns in many microorganisms, including *S. aureus*, by opsonizing them [225]. Indeed, MBL-deficient mice are susceptible to *S. aureus* infection [226]. The complement system plays an important role in microbe opsonization [136,227], as gram-positive bacteria have developed evasion mechanisms to escape this type of host defense (as reviewed by [228]).

Neutrophils are a crucial part of the immune response against *S. aureus*, as depletion of those cells increases pro-inflammatory cytokine levels and the severity of *S. aureus* infection in mice [229]. Upon pathogen recognition, neutrophils have their killing machinery activated by means of phagocytosis, releasing neutrophil extracellular traps (known as NETs), or degranulating. Phagocytosis activates the production of RNS and ROS, such as nitric oxide (NO), which are important for killing activity. Indeed, for instance, the inhibition of nitric oxide synthase (NOS) results in aggravation of sepsis and septic arthritis [230,231]. Superoxide and derived ROS are produced during a process known as respiratory or oxidative burst by NADPH oxidase [232]. The multi-subunit enzyme complex NADPH oxidase is composed of a heterodimer of gp91phox and p22phox, known as cytochrome b558, which is the catalytic core and electron transferase, and the cofactors p47phox, p40phox, p67phox, Rap1A, and a small G protein Rac2 [216,232]. Before neutrophils are activated, these subunits are disassembled: flavocytochrome b558 and Rap1A are found in membrane compartments, while p40phox, p47phox, and p67phox are located in cytosolic compartments. Even though superoxide anion by itself does not possess strong microbicidal activity, it is a precursor to other oxidants including hydrogen peroxide (H_2_O_2_), hydroxyl radical (OH^•^), hypochlorous acid (HOCl), peroxynitrite (ONOO^•^), and stronger oxidants that contribute to *S. aureus* killing [233]. In fact, as a mechanism of immune evasion, *S. aureus* induces the production of the anti-inflammatory metabolite itaconate by neutrophils, which inhibits respiratory burst and, consequently, bacterial clearance [234].

In parallel with ROS production, neutrophils generate granules that fuse with the phagosome, forming the phagolysosome. Primary granules, also called azurophilic granules, contain myeloperoxidase (MPO), an enzyme responsible for catalyzing a reaction between chloride and H_2_O_2_, resulting in HOCl, an effective antimicrobial agent that attacks microbial enzymes essential for energy production and preservation of structural integrity [235]. Patients with MPO deficiency are still able to promote microbe killing, compensating for the lack of MPO with other mechanisms, but although pathogen engulfment is efficient, killing is delayed [236]. The fusion of primary granules with the phagosome also enriches the phagosomal compartment with elastase, lysozyme, α-defensins, cathepsins, proteinase-3, and azurocidin [237]. Some of the mechanisms used by antimicrobial peptides include pore-forming, restriction of the synthesis of nucleic acids and proteins, cell cycle interruption, as well as DNA, RNA, and organelle degradation (as reviewed by [238]).

Neutrophils can also release intracellular-stored granules by exocytosis, or degranulation, to the exterior of the cell, which is dependent on cytoskeleton reorganization and changes in intracellular Ca^2+^ levels [239]. Despite their importance during extracellular bacterial clearance, uncontrolled degranulation can damage host tissue significantly. Phenol-soluble modulins secreted by *S. aureus* are known to induce neutrophil degranulation [240], and proline-rich kinase 2 (Pyk2) seems to be required for efficient clearance of *S. aureus* infection, at least in part, due to its role during the neutrophil degranulation process [241].

A third mechanism used against microorganisms, including *S. aureus*, during neutrophil defense, is the release of weblike structures called NETs. The extrusion of these structures, composed of primary granule- and cytosolic proteins, decondensed chromatin, and bound histones, is known as NETosis. NETs trap microorganisms, preventing their spread and promoting clearance [242]. Moreover, NETs are also triggered by non-infectious stimuli, such as microcrystals, immune complexes, cytokines, and alkaline pH [243,244,245]. It was previously described that neutrophils responding uniquely to *S. aureus* via NET formation did not require neutrophil lysis and are ROS independent [246], in contrast with other reports in which the release of NETs resulted in cell death [247,248]. In this pathway, when NETs culminate in neutrophil lysis (known as classical NETosis), the death process usually takes 2–4 h [247], while vital NET release takes 5–60 min, and neutrophils retain not only viability but many of their immune functions [246]. Depending on the stimuli, components of NET structures may vary—after simulation with C5a, for example, NETs contained mitochondrial DNA [249], while LPS results in lysosome-associated membrane protein 2 (LAMP2)-enriched NETs [250]. Among many *S. aureus* components capable of triggering NETs release, some studies point out Panton–Valentine leukocidin (PVL), a pore-forming toxin that promoted vital NETs extrusion [246,251]. Other *S. aureus* components, including leukotoxins GH (pore-forming cytolytic toxins) [252], AgrD (regulator of virulence factors expression) [253], and phenol-soluble modulin α (membrane-disturbing peptides) [254], also can generate NETosis. In addition, *S. aureus*-induced NET formation is strictly regulated and requires both TLR2 activation and complement protein C3, as TLR2- or C3-deficient mice were unable to release nuclear DNA or histones, nor perform TLR2 or C3a receptor stimulation alone during skin infections [255].

Although essential for pathogen clearance, aberrant NET formation or dysregulation of NETosis clearance is linked to several pathologies [256]. Prolonged or excessive NET formation contributes to chronic inflammation and/or autoimmunity, important factors of diseases like atherosclerosis, psoriasis, type II diabetes, rheumatoid arthritis, psoriasis, and gout arthritis (as reviewed by [257]). As several NET components exhibit tissue destructive capacity due to cytotoxic activities [258,259], respiratory dysfunction and poor outcomes are observed in *S. aureus*-infected cystic fibrosis patients, for example, as these patients show large amounts of MPO and nitrating species in their sputum [260,261]. Similarly, exaggerated NETs production provokes intravascular coagulation and tissue damage in septic mice [262,263], increases lung injury in *S. aureus*-induced pneumonia [264], and contribute to vegetation formation in *S. aureus* endocarditis [265]. Therefore, ideally, both NET structures and apoptotic neutrophils that have promoted bacterial killing should be removed properly by macrophages through a non-phlogistic process called efferocytosis [266]. In fact, NET formation also enhances the antibacterial activity of macrophages [267], another important cell type during host defense against *S. aureus*.

Summing up, neutrophils are essential for the host innate immune response against *S. aureus* infection. Their recruitment is orchestrated by host inflammatory molecules as well as by bacterial virulence factors. Phagocytosis, degranulation, and NET formation are neutrophil mechanisms. The primary goal of neutrophils is to kill the causative bacteria, *S. aureus*, in this case; however, this cell type also causes tissue damage, thus the microbicidal mechanisms are two-way responses. Finding balance is essential to protect the host.

#### 3.1.4. Mast Cells

Mast cells are granule-containing cells found in connective tissues and mucosa, deeply involved in allergic reactions by releasing the granules that contain proteases, cytokines, and proteoglycans, and act as antigen-presenting cells. Exacerbated or inappropriate mast cell activation is linked to several diseases, such as asthma, atopic dermatitis, and psoriasis [268].

Patients affected by atopic dermatitis and infected with *S. aureus* present a higher number of mast cells with a consequent Th1 response development and the upregulation of IFN-γ [269]. When mast cells sense intracellular *S. aureus*, the IFN-γ-mediated type 1 response promotes autocrine feedback, stimulating a cell-autonomous antimicrobial state of mast cells [270]. To control the exacerbated response, mast cells can activate Tregs via IL-2 production during chronic allergic dermatitis [271]. The staphylococcal enterotoxin B (SEB) activates TRPV1^+^ sensory neurons, which produce substance P, that in turn stimulates mast cell degranulation in skin inflammation [272].

*S. aureus* can induce mast cell activation directly. Nakamura et al. (2013) demonstrated that *S. aureus*-derived δ-toxin is a potent inducer of mast cell degranulation in allergic skin diseases [51]. The same toxin present on the skin increases epicutaneous sensitization to food allergens in mice [273]. In addition, LTA promotes skin mast cell proliferation and maturation via stem cell factor production from keratinocytes [274]. Moreover, PGN-stimulated mast cells (via TLR2) produce IL-6, IL-5, TNF-α, IL-13, and IL-4 [275]. Biofilm-secreted factors also induce mast cell infiltration and mucosal damage in chronic rhinosinusitis [276].

In summary, mast cells and their granules are present during Th1 responses, and IFN-γ produced by those cells helps mount an efficient antimicrobial response against *S. aureus*. Bacterial components, such as δ-toxin, LTA, and peptidoglycans, stimulate mast cell activation.

#### 3.1.5. Eosinophils

Eosinophils are classically known as effector cells primarily involved in parasite infections and hypersensitivity diseases, releasing inflammatory mediators, granule proteins, and lipids. Interestingly, studies have pointed out the physiologic role of eosinophils as well, serving as multifunctional leukocytes with immunoregulatory functions [277,278].

Patients with airway inflammation and infected with *S. aureus* recruit eosinophils to form extracellular eosinophilic traps (EETs) as part of the host immune defense [279]. Furthermore, serum IgE levels specific to SEB could contribute to eosinophil activation and IgE production in adult asthmatics [280]. Detection of SEB was confirmed in patients with different subtypes of chronic rhinosinusitis, acting as superantigens in the sinonasal mucosa [281]. The levels of enterotoxins-IgE were associated with disease severity, eosinophilic inflammation, and increased incidence of asthma comorbidity in *S. aureus*-associated chronic rhinosinusitis individuals [282]. Indeed, mice exposed to intranasal administration of SEB showed worse airway inflammation by increased eosinophil infiltration and production of chemokines and adhesion molecules that facilitate eosinophilic migration to the lungs [283]. During skin inflammation, *S. aureus* epicutaneous exposure resulted in eosinophil infiltration where these cells had a comparable contribution to the skin inflammation as T cells, in an eosinophil-derived IL-17A and IL-17F dependent manner [284]. Therefore, those studies point out eosinophils’ presence as aggravating factors during some *S. aureus* infections, but their role in other disease-specific conditions remains unclear.

### 3.2. Adaptive Immune Response

#### 3.2.1. T Cells

T cells, also called T lymphocytes, express a unique receptor, T cell receptor or TCR, that detects and responds to infected cells and are divided into αβ or γδ TCRs [285]. T helper (Th) cells, or CD4^+^ T cells, express membrane CD4 and recognize antigens presented by MHC class II, while cytotoxic T cells, or CD8^+^ T cells, express CD8 and recognize antigens presented by MHC class I [285]. Naïve CD4^+^ T cells can differentiate into subsets: Th1, Th2, Th9, Th17, Treg, and Tfh, based on the expression of signature cytokines and displaying different functions [286]. The Th1 subset induces type 1 responses to combat intracellular pathogens (e.g., bacteria, viruses, protozoa) by inducing macrophage polarization towards the M1 phenotype. Type 2 responses, marked by recruitment of eosinophils, basophils, mast cells, and macrophage polarization towards an anti-inflammatory profile, are elicited by Th2. Th9 cells, which differentiate in the presence of IL-4 and TGF-β, secrete IL-9 and are involved in allergic diseases [287,288,289]. Th17 mediates type 3 response to offer protection against fungi and extracellular bacteria, recruiting neutrophils to the infection site and stimulating secretion of anti-bacterial molecules by barrier tissues [290,291]. Follicular T helper cells, Tfh, help B cells produce antibodies [292], and Tregs are regulatory T cells that have the essential role of suppressing excessive immune responses and avoiding reactions to self-antigens [293].

The role of T cells during *S. aureus* infections was investigated by several studies. Lin and colleagues report that Th1 and Th17 responses have a protective role by favoring microbial killing through IFN-γ, IL-17, and neutrophil chemotaxis against *S. aureus* and *Candida albicans* [294]. Furthermore, vaccinated mice that developed moderate Th1 immunity showed better survival rates [295], and Th1-inducing vaccines reduced *S. aureus* burden significantly [296]. Blocking *S. aureus*-induced IL-10 production (which impairs T cell responses) improved bacterial clearance during subsequent systemic and subcutaneous infection [297]. Production of IL-17 is also carried out by γδ T cells, and this cell population is linked to several beneficial roles in different contexts—deficiency of γδ T cells results in impaired neutrophil recruitment and bacterial clearance in *S. aureus*-induced pneumonia [298]; similar results were observed during skin infections [299,300,301] and peritonitis [302]. A population of IL-17-producing memory γδ T cells expands upon the first *S. aureus* infection, which makes IL-1 signaling dispensable upon a second challenge [302]. Furthermore, during implant-associated infections, the chemokine receptor CCR2 induces γδ T cells in draining lymph nodes, preventing *S. aureus* spread [303].

In some contexts of *S. aureus* infection, however, T cells show a detrimental role. Depletion of CD4+ cells resulted in less tissue damage in staphylococcal arthritis [304] and early MRSA pulmonary infection [305]. In a mouse model of septic arthritis, NF-κB activation as a consequence of the Th17 response was correlated with augmented RANKL expression and bone resorption [306]. αβ T cells-deficient mice exhibit fewer bacteria recovered and less inflammation in wound tissues, which appears to be explained by modulation of CXCR2 expression and subsequent neutrophil migration to the wound tissue [307].

In conclusion, each T cell subtype can show different roles in different diseases or contexts, as they influence many other cell types, inflammatory mediators, and receptor expression depending on the circumstances.

#### 3.2.2. B Cells

The B cell-mediated immune response against *S. aureus* involves producing antibodies targeting specific antigens of the bacteria. These antibodies are crucial for opsonizing *S. aureus* and promoting its ingestion by phagocytes, such as neutrophils and macrophages, through antibody-mediated and complement-mediated mechanisms. In addition, antibodies can target virulence factors (e.g., SpA, superantigens [SAgs]), toxins (e.g., α-toxin), cell wall components (e.g., wall teichoic acids [WTA]), and non-protein antigens (e.g., lipoteichoic acid, peptidoglycans) [308] and interfere with their detrimental effects.

Naïve B cells differentiate into IgM-producing plasma cells. To produce high-affinity antibodies of other classes, most B cells need assistance from T cells recognizing the same antigen. The type of memory helper T cells involved directs the immunoglobulin class switch: Th1 responses induce IgG subclasses that can bind to Fc receptors and activate complement, Tregs promote a class switch to IgA, and Th2 cells drive IgE responses (characteristic of allergic reactions) [309]. Marginal zone (MZ) B cells, which rapidly respond to bacterial invasion by producing antibodies, regulate the splenic neutrophil accumulation during the early phase of systemic *S. aureus* infection, and interaction with neutrophils aids MZ B cells in differentiating into IgM-secreting cells and helps clear systemic bacterial infections [310]. Also, evidence shows that immune memory against *S. aureus*, indicated by high titers of class-switched antibodies, protects against severe disease. Children with low serum levels of antibodies targeting α-toxin are more susceptible to skin infections caused by MRSA [311] and tracking the fate of patients with *S. aureus* bacteremia, authors found that those who progressed to sepsis had lower anti-toxin antibody titers at diagnosis compared to those who recovered without complications [312]. Previous studies demonstrate that passive transfer of antibodies targeting various staphylococcal antigens (e.g., ClfA, Hla, IsdA, IsdB, FhuD2) provided protection against *S. aureus* in animal models [313,314,315]. Additionally, immunization with a peptide that mimics lipoteichoic acid induces memory B cells in BALB/c mice, increasing the percentage of plasma cells and memory B cells in the spleen and bone marrow. It also raises the levels of IL-6 and IL-21, two essential cytokines for memory B cell development [316].

The efficacy of antibody-mediated protection against *S. aureus* is, however, controversial. Vaccines that showed promising results in pre-clinical or even phase I, II, and III clinical trials have failed to promote satisfactory immunity in humans (summarized in [317]). In addition, the pathogen’s numerous immune evasion mechanisms challenge the efficacy of antibody-mediated defenses [318,319]. For example, factors like SpA and Sbi can block circulating antibodies, SpA induce B-cell depletion, or undermine downstream processes mediated by antibody binding to complement components [320]. This anti-humoral arsenal explains, in part, why natural exposure to *S. aureus* does not protect the host from infection. Therefore, the failure of some *S. aureus* vaccines may be attributed to the bacterium’s phenotypic variability, allowing it to switch off toxins, capsules, and adhesins during different growth phases, local environments, and in response to host defenses [317]. Adding suitable adjuvants to these identified epitopes can be crucial in designing an effective vaccine against *S. aureus* infection. Recently, bioinformatics tools have been used extensively to identify appropriate epitopes for vaccine formulation [317].

### 3.3. Other Cell Types

Besides immune cells, other cell types respond to *S. aureus*, modulating immune response and sometimes resulting in neuronal activation, pruritus, and/or pain, characteristic of some infections.

#### 3.3.1. Tissue Barrier Cells

Keratinocytes, the main cell type in the epidermis, accumulate *S. aureus* inside their lysosomes and produce IL-1α via TLR9 during AD [321]. Furthermore, there is an increase in IL-36α levels along with IL-4, which triggers B cell IgE class-switching and plasma cell differentiation [322]. Both L-1α and IL-36α, as well as IL-17 production by γδ T cells and neutrophil infiltration, are elicited by the phenol-soluble modulin (PSM)α virulence factor [323]. In fact, by co-culturing keratinocytes and neutrophils, Focken and Schittek (2024) [324] showed that neutrophil survival was enhanced by IL-8 signaling, as well as their priming to further antimicrobial activity against *S. aureus* [324]. Other inflammatory cytokines released by keratinocytes responding to *S. aureus* include IL-1β and IL-33 [325,326], the latter being a mediator of itch [327]. Interestingly, tissue-resident Langerhans cells produce IL-1β in response to *S. aureus* but not *S*. *epidermidis,* differentiating commensal from pathogenic bacteria via this specific cytokine [328]. Evidence shows that *S. aureus*-derived LTA induces damage to the skin barrier by enhancing neutrophil infiltration and IL-1 family levels and decreasing filaggrin and loricrin expression, barrier proteins required for healthy epidermis [329].

Using epithelial cells from human nasal tissue explants, authors demonstrated that IL-33 and thymic stromal lymphopoietin (TSLP) is produced during chronic rhinosinusitis with nasal polyps, perpetuating airway Th2 inflammation response [330]. Release of TSLP is induced, at least in part, by *S. aureus*-derived diacylated lipopeptide signaling through TLR2/TLR6 in keratinocytes [331]. Activation of TLR2 by peptidoglycan from *S. aureus* elicits upregulation of IL-6, TNF-α, IL-31, IL-33, ST2, and CXCL1/2 in dry skin and psoriasis models in mice [332].

During *S. aureus* skin and soft tissue infection (SSTI), Hla is secreted to disrupt epithelial barriers [333]. Interestingly, there is an estrogen-specific response to Hla [334], and G protein-coupled estrogen receptor activation limits *S. aureus* pathogenesis by reducing expression of the Hla receptor, ADAM10, on keratinocytes, as well as protecting the permeability barrier by enhancing intercellular junction integrity [335].

Altogether, this body of evidence shows how epithelial cells developed different mechanisms to limit pathogen invasion and proliferation, and communication between epithelial cells and leukocytes is essential for a proper immune response.

#### 3.3.2. Neurons and Glial Cells

As neurotransmitters and neuropeptides affect immune cells [336,337], growing evidence points out the significant role of neuroimmune communication, underlying the complex interactions in many tissues and contexts [338,339]. In response to infections, neurons produce trophic factors that recruit microglia and astrocytes which can amplify immune responses. If the inflammatory process is prolonged, persistent recruitment of effector cells builds a feedback loop that supports inflammation and may result in neuronal injury [340]. Additionally, bacteria-derived toxins can interact with the nervous system directly [341,342], and interestingly, peripheral nerve damage makes individuals more susceptible to *S. aureus* infections [343,344].

The spleen and lymph nodes are innervated by sympathetic nerve fibers [345], which control the production of proinflammatory mediators associated with type 1 responses [346,347,348]. Recently, it was demonstrated that ablation of sympathetic nerve fibers impairs the development of protective immune memory to *S. aureus* infection in mice [349], highlighting the importance of functional neural circuits for the host’s defense.

Cutaneous TRPV1^+^ expressing neurons directly sense noxious stimuli [350] and stimulate IL-23/IL-17 production, driving Th17 response during psoriasis-like inflammation [351]. Optogenetic stimulation of TRPV1^+^ sensory neurons can trigger an inflammatory reflex arc that augments host defense to *S. aureus* by providing Th17 immunity [352]. In the skin, *S. aureus* triggers CGRP release by TRPV1^+^ neurons, which in turn abrogates neutrophil influx and inflammatory macrophage phenotype, favoring anti-inflammatory macrophages instead [156]. TRPV1^+^ dorsal root ganglia (DRG) neurons respond to formyl peptides present in *S. aureus*, suggesting the direct signaling between bacteria and neurons inducing pain independently of immune cells [157]. In addition, depletion of Nav1.8-lineage neurons abolished mechanical and thermal hyperalgesia in *S. aureus* infection, indicating that this neuronal population is essential for pain behavior induced by this microorganism in mice [157]. Those animals also showed an increased local influx of neutrophils and monocytes as well as lymphadenopathy [157], and signs of augmented inflammation upon Nav1.8^+^ neuron deletion. Similarly, on a murine model of lethal *S. aureus* pneumonia, TRPV1 sensory neurons suppress neutrophil recruitment and pulmonary γδ T cell-mediated defenses, worsening the condition. Neuron ablation increased survival rates, cytokine levels, and lung bacterial clearance [353].

The skin is also innervated by other neuronal subsets, including nonpeptidergic MrgprD-expressing sensory neurons, involved in itch sensation, inflammatory pain, and mechanical stimuli [354,355,356]. In various cutaneous inflammation models, ablation of MrgprD-expressing neurons led to mast cell degranulation and glutamate release, emphasizing the suppressive function of those neurons controlling exacerbated inflammation [357]. On the other hand, this ablation also augmented the immune defenses against *S. aureus*, underlining the double-edged sword of inflammatory milieu.

*S. aureus*-associated brain abscesses develop after local infections, such as in paranasal sinuses, spread via blood circulation, or direct invasion in cases of head trauma [358,359]. In addition to leukocyte recruitment, microglia induce upregulated expression of TNF-α, IL-1β, IL-12, MCP-1, and macrophage inflammatory protein-2 (MIP-2) [360,361]. Again, at the same time those mediators helped control infection, surrounding tissue damage was observed [362]. In this sense, minocycline was found to reduce bacterial burden while suppressing microglial/astrocyte activation and IL-1β and CCL2 levels, being an interesting therapeutic approach to treat brain abscesses [363].

**Figure 3:**
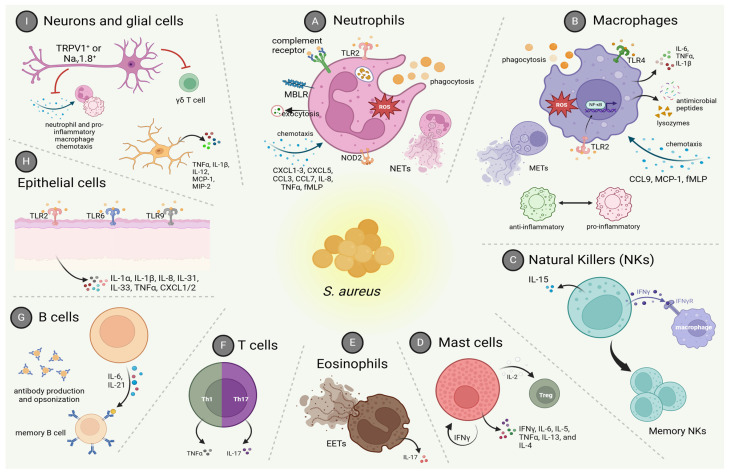
How different immune and non-immune cells respond to *S. aureus* infection. (**A**) Neutrophils are attracted (chemotaxis) by IL-8, CXCL1, CXCL2, CXCL3, CXCL5, CCL3, CCL7, TNFα, and formylated peptides to the site of infection [207,208,209,210,211,212]. Neutrophils recognize *S. aureus* through their pattern recognition receptors (e.g., TLR-2), NOD-like receptors (NOD2), complement receptors, and Mannose-binding lectin receptors (MBLR), favoring opsonization and phagocytosis [220,221,222,223,224,225,227]. Besides phagocytosis, neutrophilic killing machinery includes ROS production, release of NETs, and degranulation [233,235,237,239,242,251,252,253,254]. (**B**) Macrophages chemotaxis molecules include CCL9, MCP-1, and fMLP [132,133,138], and upon bacterial encounter and recognition through TLR2/4 [142,143,149] those cells are polarized to either pro-inflammatory (to enhance antimicrobial activities) or anti-inflammatory (as an immune evasion mechanism exerted by S. aureus) [141,150,151,152,153,154,155]. Downstream antimicrobial mechanisms of macrophages include NF-κB activation, ROS generation, release of cytokines, antimicrobial peptides and lysozymes, and METs [167,168,171,172,173,175,183]. (**C**) Natural killers (NKs) produce IFN-γ to activate macrophages for a robust antimicrobial response [189], play a critical protective role through IL-15 signaling [192], and turn into long-lived memory-like cells for faster response in the future [194]. (**D**) Mast cells promote IFN-γ-mediated type 1 response via autocrine feedback [270], activate Tregs via IL-2 [271], and produce IL-6, IL-5, TNF-α, IL-13, and IL-4 during *S. aureus* infections [275]. (**E**) Eosinophils form EETs and contribute to skin inflammation in an IL-17-dependent manner [279,284]. **(F**) T cells differentiate into Th1 and Th17, which elicit type 1 (against intracellular bacteria) and type 3 (to kill extracellular bacteria) responses, respectively. They favor microbial killing through IFN-γ, IL-17, and neutrophil chemotaxis [294,295]. (**G**) B cells produce antibodies to opsonize *S. aureus* [308] and secrete IL-6 and IL-21, two essential cytokines for memory B cell development [316]. (**H**) Epithelial cells sense *S. aureus* through TLR2/6/9 and secrete several cytokines and chemokines to trigger and maintain an immune response [321,322,324,325,326,328,329,330,331]. (**I**) Neurons and glial cells can interact with bacteria directly, and specifically, TRPV1^+^ and Nav1.8^+^ neurons abrogate neutrophil influx, γδ T cell-mediated defenses, and inflammatory macrophage phenotype [156,157,350,353]. In turn, microglia induce upregulated expression of TNF-α, IL-1β, IL-12, MCP-1, MIP-2, and leukocyte recruitment [360,361].

Despite all those types of cells involved during *S. aureus* infection (summarized in Figure 3), in chronic or severe cases bacteria persist, and this is due to virulence strategies acquired along the way―that continue to evolve. In the next Sections, we detail the main *S. aureus* virulence factors identified so far and how they manage to evade immune response.

## 4. Virulence Factors: General Virulence Strategies

Despite being frequently associated with human diseases, *S. aureus* colonizes humans persistently in a variety of sites such as the nose, skin, throat, axillae, groin, and intestine, establishing an intricate interplay with the human body and other commensal microorganisms [364,365]. Colonization is usually harmless, although it is a risk factor for the development of opportunistic infections that can vary from mild skin and soft tissue infections to severe invasive infections, such as osteomyelitis, septic arthritis, bacteremia, pneumonia, and endocarditis (see Section 2) [13,365]. The modulation of persistence as part of the host microbiota and the potential pathogenicity requires several mechanisms of host immune evasion and virulence strategies [365]. These strategies target both innate and adaptive immune responses.

As mentioned previously, neutrophils are critical in controlling *S. aureus* infections [365]. However, *S. aureus* produces factors that inhibit neutrophil chemotaxis and killing activity [365]. For example, *S. aureus* can block neutrophil receptors and impair the host’s complement system [365,366]. *S. aureus* can produce proteins that prevent opsonization and phagocytosis [367] and modify cell surface charges to repel antimicrobial peptides [368,369]. Furthermore, *S. aureus* can produce leukocidins that form pores in neutrophil membranes, leading to cell lysis [370].

Biofilm formation is another strategy that represents a challenge in managing *S. aureus* infections since it enhances antibiotic resistance and immune clearance. Within biofilms, *S. aureus* evades immune detection by masking its PAMPs [365]. The biofilm matrix can also be a barrier to antibiotic penetration, contributing to chronic and recurrent infections [371].

*S. aureus* is known for its diverse array of factors and mechanisms that facilitate persistence in the host. In the following section, the most important virulence factors of *S. aureus* known to date will be discussed in detail.

### Mechanisms of Immune Evasion

*S. aureus* employs a range of tactics to evade neutrophil destruction. It prevents neutrophils from migrating from the blood to tissues, impeding their activation and movement toward infection sites [372]. Additionally, *S. aureus* obstructs phagocytosis by forming aggregates, developing protective surface structures, and creating biofilms [372]. It also blocks opsonization to avoid immune recognition and neutralizes the killing mechanisms of neutrophils [372,373]. Furthermore, *S. aureus* can directly kill neutrophils by releasing toxins or inducing apoptosis [372,373].

Neutrophils must transmigrate through a multistep cascade to finally reach the site of infection outside the circulation. These steps comprise rolling and adhesion to the endothelium, followed by diapedesis and orientation along the chemoattractant gradient [365,374]. Neutrophils are activated by cytokines and factors of the complement system and attracted by host-derived substances such as leukotriene B4 or IL-8 and bacterial components [20]. Gram-positive bacteria, like *S. aureus*, release activators and chemoattractants such as N-terminal lipoylated structures of lipoproteins [375], peptidoglycan [376], unmethylated CpG DNA sequences [377], and formylated peptides [378]. The former group is collectively identified as PAMPs, and they are responsible for the activation of TLRs [379].

**Figure 4:**
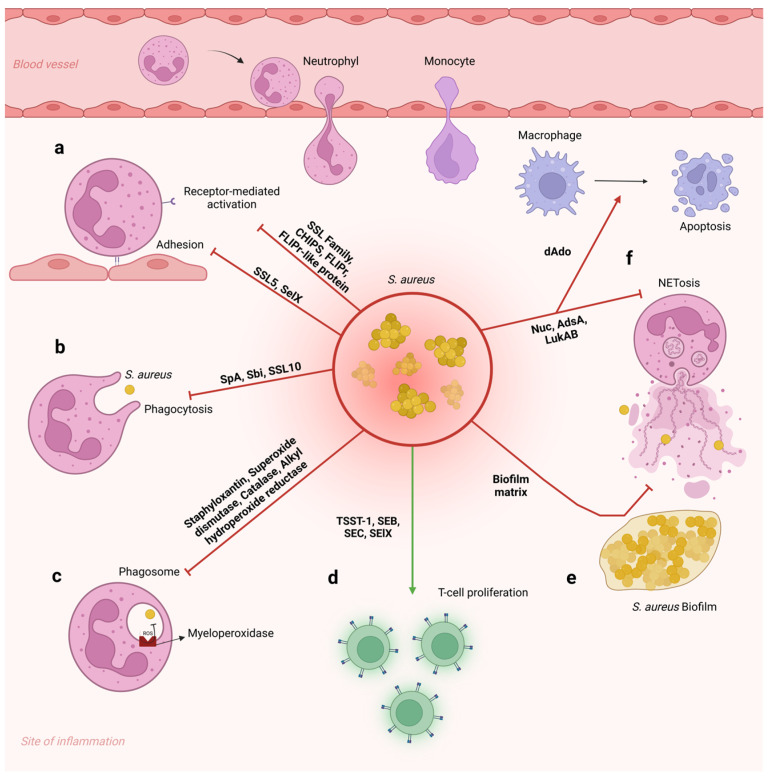
*Staphylococcus aureus* immune evasion strategies. (**a**) *S. aureus* hinders neutrophil adhesion and receptor-mediated activation, thereby blocking their migration to infection sites. This process is mediated by virulence factors such as staphylococcal superantigen-like (SSL) family proteins, chemotaxis inhibitory protein of *S. aureus* (CHIPS), FPRL1 inhibitory protein (FLIPr), FLIPr-like protein, and staphylococcal enterotoxin-like toxin X (SElX) [138,365,384,391]. (**b**) To evade phagocytosis by neutrophils, *S. aureus* utilizes surface proteins like surface protein A (SpA), *S. aureus* binder of IgG (Sbi), and SSL10, which interfere with the host immune response [320,392,393,394]. (**c**) Inside the phagosome, *S. aureus* neutralizes reactive oxygen species produced by myeloperoxidase (MPO) to resist neutrophil-killing mechanisms. This is achieved through enzymes such as staphyloxanthin, superoxide dismutase, catalase, and hydroperoxide reductase [365,405,406,407,408,409,410]. (**d**) Superantigens produced by *S. aureus*, including toxic shock syndrome toxin-1 (TSST-1), staphylococcal enterotoxin B (SEB), staphylococcal enterotoxin C (SEC), and SElX, cause excessive T-cell proliferation, leading to immune dysregulation and potential tissue damage [365,398,420]. (**e**) *S. aureus* forms biofilms that provide a protective matrix, enhancing resistance to the host immune system [20,152]. (**f**) *S. aureus* toxins, such as staphylococcal nuclease (Nuc), adenosine synthase A (AdsA), and leukocidin AB (LukAB), inhibit neutrophil extracellular trap (NET) formation (NETosis) [420,421]. Nuc and AdsA also induce macrophage apoptosis, impairing immune clearance of the bacteria [365].

*S. aureus* blocks neutrophil extravasation, activation, and chemotaxis by numerous factors and mechanisms. *S. aureus* produces staphylococcal superantigen-like (SSL) proteins with various immunomodulatory functions, including inhibition of chemotaxis and phagocytosis (Figure 4). However, their full range of activities is unknown [20]. SSL consists of fourteen exoproteins; eleven of these are encoded on the staphylococcal pathogenicity island 2 (SaPI 2), present in all strains of *S. aureus*, while the other three are in the immune evasion cluster [380,381]. These molecules were identified based on sequence and structural similarities to superantigens, though they lack superantigenic activity [382]. SSL family members inhibit activation and chemotaxis through diverse mechanisms. For example, staphylococcal superantigen-like 5 (SSL5) binds to P-selectin glycoprotein ligand-1 (PSGL-1), inhibiting neutrophil adhesion and extravasation [383]. SSL5 and SSL1 also inhibit matrix metalloproteinases, reducing neutrophil motility and chemotaxis [384]. More recently, it was discovered that SSL1 exhibited in vitro protease activity. Also, protease activity was observed in a rabbit ocular infection model since SSLs are not expressed in mouse infection models [365,385]. SSL7 has been found to bind C5 and immunoglobulin A (IgA), thereby inhibiting the complement system and blocking FcαRI binding [386]. SSLs are challenging to study because they are not expressed in mouse infection models, but overexpression vectors have shown that SSL3 contributes to staphylococcal pathogenesis by inhibiting toll-like receptor 2 [387]. SSL13, however, seems to induce neutrophil responses, highlighting the complex interactions of immune-modulating molecules during *S. aureus* infection [388].

Chemotaxis inhibitory protein of *S. aureus* (CHIPS), FLIPr, FLIPr-like, and staphopain protease also inhibit chemotaxis (Figure 4). CHIPS acts as a potent and specific inhibitor of neutrophil and monocyte chemotaxis toward C5aR and formyl peptide receptor 1 (FPR1) [138,389], while FLIPr and FLIPr-like inhibit FPR1 and FPR2 [390,391]. This inhibition prevents neutrophil migration toward the infection site by obstructing the chemotactic signals necessary for recruitment [138,390]. For example, FLIPr impairs neutrophil responses to FPR1 agonists and exerts anti-inflammatory activity by inhibiting calcium mobilization and cell migration toward chemoattractants [390]. Specifically, it inhibits calcium mobilization in neutrophils stimulated with various molecules, including MMK-1, WKYMVM, prion-protein fragment PrP 106–126, and amyloid β 1–42 [390]. FLIPr also prevents neutrophil-directed migration toward MMK-1 and partially toward fMLP. It binds to FPRL1 and, at higher concentrations, to FPR [390].

In addition to chemotaxis inhibition, *S. aureus* employs other strategies to evade the immune system, notably by interfering with phagocytosis. Effective phagocytosis requires the bacterial targets to be opsonized with antibodies or complement. *S. aureus* interferes with opsonization using proteins such as surface protein A (SpA), which binds nonspecifically to immunoglobulins (Igs) [392], creating a “camouflage” effect, or the *S. aureus* binder of IgG (Sbi), and SSL10, which prevents receptor-mediated phagocytosis [320,393,394] (Figure 4). *S. aureus* also produces proteins that inhibit the complement system, such as staphylococcal complement inhibitor (SCIN), which disrupts complement pathways inhibiting C3 convertases [395], and others like extracellular fibrinogen-binding protein Efb, extracellular complement-binding protein Ecb, and SSL7, which inhibit specific complement pathways or components [386,396,397].

Another virulence factor includes superantigens (sAgs), bacterial toxins that stimulate T cell proliferation and disrupt immune regulation, thereby enhancing invasive *S. aureus* infections and colonization [365] (Figure 4). They bind to various variable-β chains in the T cell receptor. Well-known examples include toxic shock syndrome toxin-1 (TSST-1) and staphylococcal enterotoxins B and C, but over 26 sAgs facilitate binding to many variable-β chains in the T-cell receptor [365,369,398].

Recently, staphylococcal enterotoxin-like toxin X (SElX) was found in 95% of *S. aureus* isolates examined [365]. SElX stimulates T cell activation, promoting necrotizing pneumonia in a rabbit model, and hinders phagocytosis by binding to neutrophils through surface receptors [399] (Figure 4). This dual role in disrupting innate and adaptive immune responses highlights its significance. Genomic studies have also identified staphylococcal sAgs SElW in 97% of the isolates [398], with the gene-encoding SElW being truncated in some staphylococcal clones but full length in others. SElW exhibits strong T cell activation in a bacteremia model, specifically driving T cell mitogenic activity in the human and livestock-adapted CC398 clone [365,398].

A broad array of molecules helps *S. aureus* survive after being phagocytosed, where it encounters toxic products like reactive oxygen species, nitric oxide, antimicrobial peptides, and neutrophil serine proteases (NSPs) [365,400,401]. The extracellular adherence protein (Eap) and its homologs (EapH1 and EapH2) can inhibit NSPs at low concentrations, preventing bacterial killing, regulation of neutrophil extracellular traps (NETs), and degradation of phenol-soluble modulins (PSMs) [400,401]. PSMs target the neutrophil formyl-receptor 2, causing degranulation and neutrophil lysis [401]. NSPs can degrade and inactivate various staphylococcal immune evasion factors, including CHIPS, though they act differently on homologous proteins, potentially explaining the redundancy of these factors [365,366]. Eap proteins protect against NSP activity on staphylococcal immune evasion factors [401,402].

Inside the phagosome, myeloperoxidase (MPO) utilizes hydrogen peroxide to produce reactive oxygen species, collaborating with intracellular proteases to combat *S. aureus* [403,404]. High-throughput screening identified a factor that inhibits neutrophil killing by binding and inhibiting MPO, named staphylococcal peroxidase inhibitor [405] (Figure 4). This molecule’s expression increases after phagocytosis, evading MPO-dependent killing [405]. Similar to other *S. aureus* immune evasion factors, the staphylococcal peroxidase inhibitor does not interact with or inhibit MPOs from other species [365]. *S. aureus* neutralizes reactive oxygen species (ROS) using staphyloxanthin, superoxide dismutase, catalase, and alkyl hydroperoxide reductase [365,406,407,408,409,410] (Figure 4). It inhibits oxidative bursts by converting ADP and AMP to adenosine and resists copper-induced ROS production [411]. *S. aureus* also neutralizes antimicrobial peptides (AMPs) by altering the bacterial surface charge, degrading AMPs, and reducing recognition of AMPs [412].

*S. aureus* expresses various cell-wall-anchored adhesins, including those in the MSCRAMM (microbial surface components recognizing adhesive matrix molecules) family [413]. The serine-aspartate repeat protein D (SdrD) is a significant MSCRAMM protein contributing to colonization, infection, and abscess formation [414,415]. It inhibits the innate immune killing of *S. aureus* and promotes bacterial survival in human blood. However, its mechanisms remain unclear [416]. Another serine–aspartate repeat protein, SdrE, binds complement factor H, aiding in complement evasion [417]. The fibronectin-binding protein FnbpB plays a critical role in binding to host ligands, activating platelets, and invading non-phagocytic cells [418]. It also binds plasminogen, which can be converted to plasmin by host activators or staphylokinase, and has a novel role in binding histones, leading to their degradation [419].

Host deployment of NETs is an effective defense against bacterial intrusions, leading to neutrophil death (NETosis) [365]. *S. aureus* counters this by secreting staphylococcal nuclease (Nuc) to degrade the NETs DNA backbone and using adenosine synthase A (AdsA) to convert extracellular ATP and ADP into adenosine, an anti-inflammatory molecule [420,421] (Figure 4). AdsA also converts dAMP into deoxyadenosine (dAdo), triggering apoptosis in macrophages and thereby reducing their activity at the infection site [365] (Figure 4).

Another crucial group of *S. aureus* immune evasion molecules is the bicomponent pore-forming toxins (leukocidins), which are primarily associated with neutrophilia but also target various immune cells [365,422]. Leukocidins create octameric membrane-spanning pores, leading to cell lysis by binding to specific cell receptors [365]. LukAB, a leukocidin, kills dendritic cells, preventing antigen presentation necessary for adaptive immunity [423]. Additionally, *S. aureus* produces toxins such as α-toxin, which is significant for its cytolytic properties [424].

Biofilm formation is another factor in *S. aureus* pathogenesis, on top of the host-evasion factors previously discussed. These biofilms can form on non-living materials of indwelling medical devices and tissue surfaces, such as heart valves in endocarditis [425]. Infections linked to biofilm growth on biomedical devices like catheters and implants or host tissues such as wounds and heart valves prove challenging to treat [425]. Biofilm formation progresses through three main stages: adhesion, maturation/proliferation, and detachment [20]. They protect the bacteria by embedding them in extracellular matrices of proteins, polysaccharides, and extracellular DNA [425]. Additionally, the secretion of factors like hemolysins, nucleases, and PSMs supports effective host evasion in the biofilm lifestyle, which might promote the shifting of the immune response toward an anti-inflammatory state [365,426].

The primary role of biofilm formation during infection is to protect the bacteria from phagocyte attacks [20,152] (Figure 4). Some argue biofilm formation and abscesses share similar characteristics, but critical differences exist [20]. For instance, biofilms are typically not surrounded by large layers of neutrophils as abscesses are, likely because *S. aureus* in biofilms exists in a less aggressive state and does not produce or shed a significant number of chemoattractant molecules through the biofilm matrix [427]. Furthermore, *S. aureus* biofilms have been demonstrated to alter the host immune response, promoting an anti-inflammatory state [20,152,427].

Several mechanisms regarding the induction of an anti-inflammatory state in the human immune system by *S. aureus* biofilms can be highlighted. For example, leukocidins PVL and HlgAB help *S. aureus* survive in biofilms by triggering NET formation, which traps and kills planktonic bacteria [365,370]. Bacterial DNase (Nuc) also digests NET DNA, aiding biofilm dispersal and increasing metastasis infection [428]. Moreover, LukAB assists in phagocyte escape within biofilms [428]. *S. aureus* biofilms modulate the host immune response to an anti-inflammatory state, recruiting myeloid-derived suppressor cells (MDSCs) and polarizing macrophages to M2-like anti-inflammatory states [365]. MDSCs inhibit T-cell activation and promote bacterial persistence [429]. Activated macrophages typically promote inflammation through immunometabolism, with metabolites like succinate (pro-inflammatory) and itaconate (anti-inflammatory) [365]. *S. aureus* biofilms, however, induce macrophages to an anti-inflammatory status, switching their metabolism to mitochondrial oxidative phosphorylation (OXPHOS) [155]. *S. aureus* biofilms produce IL-10 via staphylococcal lactate, reflecting the interaction of bacterial and host metabolism [430]. Furthermore, host itaconate inhibits bacterial glycolysis, promoting biofilm formation [431]. Table 6 summarizes the key immune evasion mechanisms employed by *S. aureus*, highlighting the primary proteins and factors involved in targeting various aspects of the host immune response.

*S. aureus* produces diverse virulence factors that enable its persistence and pathogenicity, manifesting in both mild skin infections and severe conditions like pneumonia and endocarditis. It is crucial to comprehend the mechanisms underlying these virulence factors and how the host responds to them, as this knowledge is vital for devising effective therapeutic strategies and treatments.

## 5. Future Perspectives

*S. aureus* is the leading cause of hospital-acquired infections and can cause a wide range of inflammatory diseases and pain. In this article, we present the most recent knowledge about *S. aureus*, addressing the main inflammatory diseases caused by this bacterium, its pathogenic mechanisms, and the main cell types involved in the host immune response upon an infection. A better understanding of these aspects can guide the development of new targeted therapies for treating diseases and pain induced by *S. aureus*.

Despite the fact that *S. aureus* is one of the most extensively studied microorganisms, there is still a significant gap in knowledge, unfortunately. Regarding the pathogenicity of *S. aureus*, some mechanisms remain largely unknown. Several inflammatory cells are involved in the pathogenesis of this bacterium, including Th9, Tfh, and ILC cells, whose mechanisms remain open for investigation [15]. Studies demonstrate that Th9 cells are important regulators of immunity to autoimmune diseases [432] and tumor immunity [433], in addition to acting in the pathophysiology of respiratory tract allergies, such as asthma [434] and allergic rhinitis [435]. However, despite there being data in the literature that correlate the role of these cells with the development of several diseases, little is known about the mechanisms of *S. aureus* involved in the development of a Th9-type inflammatory response. In the case of Tfh and ILC cells, some studies have associated them with the pathogenic mechanisms of *S. aureus* [436,437,438]; however, there is still a need for further investigation of the involvement of these cells in the inflammatory diseases caused by this bacterium.

*S. aureus* can induce different forms of cell death, which have a significant role in the pathogenesis of several diseases [15]. However, the roles of some types of cell death, such as ferroptosis (regulated and iron-dependent cell death) [439] and cuproptosis (cell death caused by excess copper and accumulation of mitochondrial proteins) [440], remain poorly defined in the context of *S. aureus* infection. Furthermore, there are few data in the literature that investigate the pathogenic mechanisms of *S. aureus* with pain present in different types of diseases. This might be of importance considering that *S. aureus* can shape the immune response by activating nociceptor neurons [157]. Thus, future research may allow greater knowledge about the mechanisms involved in pain and infection by *S. aureus*, increasing the possibility of developing new therapeutic and prophylactic approaches for diseases correlated to this bacterium. Few data in the literature address the treatment of pain present in several diseases caused by *S. aureus.* Blake et. al. demonstrated that QX-314, a membrane-impermeable sodium channel blocker, relieves pain when administered to sensory neurons. They observed that QX-314 silences neuronal activity caused by the administration of pore-forming toxins and potentially blocks all major pain modalities (spontaneous pain, thermal, and mechanical hyperalgesia) during MRSA infection [441]. Another study demonstrated that systemic blockade of CXCR2, through DF2156A (an antagonist of CXCR1 and CXCR2), decreased inflammation, pain, and tissue damage caused by *S. aureus* [442]. Thus, investing in research that addresses the pain mechanisms caused by *S. aureus* and seeking therapeutic approaches that contribute to improving patients’ quality of life is a current need that is open for investigation.

Treatment of infections caused by *S. aureus* is becoming increasingly difficult, mainly due to the acquisition of resistance genes, selection of antibiotic-resistant strains (e.g., methicillin-resistant *S. aureus*) [1], biofilm formation [443], and strategies to evade the host’s immune system [444]. These adversities, as well as others not reported here, make *S. aureus* a growing threat to global health. Thus, driven by the increasing antibiotic resistance and additional difficulties in finding functional treatments (e.g., vaccines), there is a growing interest in developing novel and/or adjuvant strategies for treating *S. aureus* infections. Table 7 summarizes some clinical and preclinical studies of promising new approaches for the treatment of diseases caused by *S. aureus* that have structural components and virulence factors as targets.

Despite enormous progress in research into *S. aureus* and its related diseases, it is still difficult to find satisfactory treatments, especially for resistant strains. Regarding vaccines, many classical methods and new technologies have been tested, and even though some research reveals vaccines with potential protective effects, none have been approved for use to date [479]. Thus, future research that addresses new methodologies, such as reverse vaccinology and bioconjugates, appears promising and may be the center of future vaccine development [444,480].

Taken together, the development of new therapeutic drugs that work according to different principles than those currently available may be the solution to reduce the factors that contribute to the establishment of resistance and spread of *S. aureus* [19]. In this context, therapeutic approaches that control the release of molecules, such as methods for incorporating microparticles and nanotechnology, are also targets of interest in future research. This controlled release minimizes systemic toxicity and reduces the induction of resistance to treatments by *S. aureus*, overcoming some of the bacteria’s evasion mechanisms and helping in the combat and prophylaxis of the infection [79].

Another avenue of research of great interest is the use of new personalized methods to combat infections, such as the Clustered Regularly Spaced Short Palindromic Repeats (CRISPR/Cas9) technique [79,481]. Bikard et al. developed programmable and specific antimicrobials through CRISPR/Cas9. This reprogrammed antimicrobial was responsible for targeting MRSA virulence genes, making it susceptible to methicillin again [482].

In this same context, bioinformatics tools can be great allies in discovering the pathogenic mechanisms of *S. aureus* and in the treatment of pain and related diseases. Several studies already use these tools for genomic characterization of new strains of MSSA [483] and MRSA [484,485], which expands the knowledge about these strains. Furthermore, Abujubara et al. reported the synthesis of a series of inhibitors of Sortase A, a surface enzyme of gram-positive bacteria, through a computational analysis. Applying these inhibitors in in vitro assays, they found that three of them inhibit the growth of *S. aureus* and the formation of biofilms [486]. In addition, Muthukrishnan et al. demonstrate the feasibility of a bioinformatics approach in using a patient’s immune proteome active against *S. aureus* to diagnose *S. aureus* musculoskeletal infections (MSKI) [487].

In summary, an advanced understanding of the pathophysiology of *S. aureus* in different types of diseases and in pain and knowledge of structural pathogenic and resistance mechanisms may aid in the discovery and development of new strategies to treat *S. aureus* infections.

## Figures and Tables

**Table 1:** Skin microbiome of patients with atopic dermatitis.

Agents		Skin Colonization in AD	Relative Abundance in the Skin in AD (%)	References
*Staphylococcus*	*aureus*	Increased	30%	[47,50]
	*capitis*	Reduced	1.9	
	*epidermidis*	Reduced	2.6	
*Acinetobacter*		Reduced	4.5	
*Bacteroidetes*		Reduced	4.3	
*Corynebacterium*		Reduced	5	
*Cutibacterium*		Increased	4.2	
*Micrococcus*		Reduced	1.6	
*Paracoccus*		Increased	4.8	

**Table 2:** Main causative agents of bacteremia and sepsis.

Agents	% Prevalence in Hospitalized Children (<14 Years)	% Prevalence of Hospital-Onset (≥18 Years)	% Prevalence of Community-Onset (18–44 Years)	Refs.
*Staphylococcus aureus*	12.7	17.5	14.0	[57,58,59,60]
*Acinetobacter baumannii*	2.6	8.7	-	
*Enterococcus faecalis*	2.2	10.4	2.3	
*Enterococcus faecium*	12.7	5.4	-	
*Escherichia coli*	26.8	19.8	28.8	
*Klebsiella pneumoniae*	20.2	22.1	7.9	
*Pseudomonas aeruginosa*	1.3	15.6	4.2	
*Salmonella* spp.	5.7	-	5.6	
*Streptococcus agalactiae*	3.9	-	1.4	
*Streptococcus pneumoniae*	11.8	0.6	2.3	
*Streptococcus* *viridans*	-	-	2.8	

**Table 3:** Causative agents of infective endocarditis.

Agents	Percentage of IE Cases	Prevalence in South America	References
*Staphylococcus aureus*	35–40%	17%	[71,72]
Coagulase-negative *staphylococcus*		7%	
*Enterococcus*	10%	8%	
Fungal	2%	1%	
HACEK	5%	3%	
Polymicrobial (≥2 microorganisms)	8%	-	
*Streptococcus bovis*	15%	7%	
*Streptococcus* *viridans*	20%	6%	

HACEK: *Haemophilus* spp., *Aggregatibacter* spp., *Cardiobacterium hominis*, *Eikenella corrodens e Kingella* spp.

**Table 4:** Main causative agents of osteomyelitis.

Agents	Susceptibility Factors	Tropism	Incidence in Skeletal Infections (%)	Age	References
*Staphylococcus aureus*	Injectable drug users, immunocompromised, spinal column surgery, orthopedic fixation devices, diabetes mellitus, vascular insufficiency, contaminated open fracture	Lower extremity prosthetic joint	20–30	Adults and children	[80,81,82]
		Trauma or fracture-related	20–40		
		Haematogenous	40		
		Foot and ankle	45–55		
*Enterobacteriaceae* spp.	Hospitalization (nosocomial source)	Lower extremity prosthetic joint	<5	Newborn babies	
		Trauma or fracture-related	5–20		
		Haematogenous	5		
		Foot and ankle	10–15		
*Pseudomonas* spp.	Injectable drug users, urinary infection, hospitalization (nosocomial source)	Lower extremity prosthetic joint	<5	Adults	
		Trauma or fracture-related	5–10		
		Haematogenous	5–10		
		Foot and ankle	10–20		
*Staphylococcus epidermidis*	Spinal column surgery, orthopedic fixation devices, diabetes mellitus, vascular insufficiency, contaminated open fracture	Lower extremity prosthetic joint	20–25	Adults and children	
		Trauma or fracture-related	5–15		
		Haematogenous	<5		
		Foot and ankle	<5		
*Streptococcus* spp.	Diabetes mellitus, vascular insufficiency, contaminated open fracture	Lower extremity prosthetic joint	<5	Adults, children, and newborn babies	
		Trauma or fracture-related	5–10		
		Haematogenous	5–10		
		Foot and ankle	5–20		

**Table 5:** Main causative agents of septic arthritis.

Agents		% Prevalence	Age	Involved Joints	References
*S. aureus*	MRCNS	8.8	Adults	Knee	[104,105]
	MSCNS		Adults	Knee and shoulder	
	MRSA	53	Children and adults	Knee, hip, and shoulder	
	MSSA		Children and adults	Knee, hip, and shoulder	
*Enterobacteriaceae*		8.3	Children	Knee and hip	
*Pseudomonas*		2.2	Children and adults	Knee and hip	
*Streptococcus*		22.1	Children and adults	Knee, hip, and shoulder	

MRCNS: methicillin-resistant coagulase-negative *Staphylococci*; MSCNS: methicillin-sensitive coagulase-negative *Staphylococci*; MRSA: methicillin-resistant *Staphylococcus aureus*; MSSA: methicillin-sensitive *Staphylococcus aureus*.

**Table 6:** Immune evasion mechanisms of Staphylococcus aureus and the main proteins involved.

Immune Evasion Mechanism	Function/Type	Key Proteins/Factors	References
Inhibition of Neutrophil Migration and Chemotaxis Inhibition of Phagocytosis Complement System Evasion	Prevents neutrophils from reaching infection sites	Staphylococcal superantigen-like proteins (SSL5 and SSL1), CHIPS, FLIPr, FLIPr-like	[383,384,389,390]
	Prevents bacteria from being engulfed by immune cells	Staphylococcal protein A (SpA), *S. aureus* binder of IgG (Sbi), SSL10	[320,392,394]
	Blocks complement activation	Staphylococcal complement inhibitor (SCIN), Extracellular fibrinogen-binding protein (Efb), Extracellular complement-binding protein (Ecb), SSL7	[386,395,396,397]
Superantigen Activity Interference with Reactive Oxygen Species (ROS)	Stimulates T cell proliferation and disrupts immune regulation	Toxic shock syndrome toxin-1 (TSST-1), Staphylococcal enterotoxins B and C (SEB, SEC), Staphylococcal enterotoxin-like X (SElX)	[365,369,398,399]
	Neutralizes ROS produced by immune cells	Staphyloxanthin, Superoxide dismutase, Catalase, Alkyl hydroperoxide reductase	[406,408,409,410]
Antimicrobial Peptide Resistance Adhesion to Host Cells and Tissues NETs Evasion Biofilm Formation	Resists AMP-mediated bacterial killing	Surface charge alteration, AMP degradation	[412]
	Promotes bacterial adhesion and evasion	MSCRAMMs, Serine-aspartate repeat protein D (SdrD), Serine-aspartate repeat protein E (SdrE), Fibronectin-binding protein B (FnbpB)	[414,417,418,419]
	Destroys neutrophil extracellular traps (NETs)	Staphylococcal nuclease (Nuc), Adenosine synthase A (AdsA), Leukocidins (LukAB)	[420,421,428]
	Protects bacteria from immune attacks	Biofilm matrix (proteins, polysaccharides, extracellular DNA), Leukocidins (PVL, HlgAB), Bacterial DNase (Nuc), AdsA	[152,365,425,426,428]
Promotion of Anti-inflammatory State	Shifts immune response to an anti-inflammatory state	Staphylococcal biofilm components, Leukocidins (PVL, HlgAB), Adenosine, IL-10	[370,429,430,431]

CHIPS: Chemotaxis inhibitory protein of *S. aureus*; MSCRAMMs: microbial surface components recognizing adhesive matrix molecules; IL: Interleukin.

**Table 7:** Promising treatments against *S. aureus* infections.

Agent (Type)	Name (Compound)	Mechanism of Action	Research Stage	Recommendation	References
Vaccine	SA4Ag (composed of CP5 and CP8 conjugated with rmClfA and rMntC)	Directed against the capsular polysaccharides of *S. aureus*. Induces high levels of antibodies that kill bacteria in adults	Clinical Stage. Phase 1 study completed. Phase 2B study in progress	Postoperative infections	([445]; ClinicalTrials.gov: NCT01364571, NCT02364596, NCT02388165)
	NDV-3 and NDV-A (composed of a recombinant form of the Als3 protein from *Candida albicans*)	Directed against the Als3 protein. Induces rapid antibody and T cell responses. In preclinical studies, increased neutrophil infiltration and expression of IL-17 and IL-22	Clinical stage. Phase 1 and 2 studies completed	*S. aureus*, *MRSA*, and *Candida albicans infections*	([446,447]; ClinicalTrials.gov: NCT01926028, NCT03455309)
	STEBVax (composed of a mutated recombinant form of SEB)	Interrupts the interaction of enterotoxin B with MHC class II receptors. Induces the production of toxin-neutralizing antibodies	Clinical stage. Phase 1 studies completed	Toxic shock syndrome and protection against a wide range of staphylococcal superantigens	[448]
	IBT-V02 (composed of seven toxoids of *S. aureus*)	Targeted against α-toxin, LukS, LukF, LukAB, SEA, SEB and TSST-1	Clinical stage. Phase 1 studies underway	Postoperative MRSA infections, skin and soft tissue infections	[449,450]
	GSK2392103A (composed of CP5 and CP8 conjugated to tetanus toxoid, a mutated α-toxin, and ClfA)	Directed against the capsular polysaccharides of *S. aureus*, α-toxin, and ClfA. Induces robust humoral immune responses	Clinical stage. Phase 1 studies completed	-	([451]; ClinicalTrials.gov: NCT01160172)
	GSK3878858A	Directed against recombinant proteins	Clinical stage. Phase 1 in progress	Skin and soft tissue infections	[449]
	GAS	Directed against the cell completely. Induces increased antibody production, increased T cell population, and decreased bacterial load	Preclinical stage	Septicemia	[452]
	*S. aureus vaccine*	Induces multifaceted B and T cell responses to *S. aureus* antigens. Induces the production of antibodies, Th1 or Th17 response	Preclinical stage	Bacteremia, dermonecrosis, skin abscess, and gastrointestinal colonization	[453]
	ABN-701 (composed by the modified SEB)	Directed against *S. aureus* enterotoxin B. It induces neutralization of antigen activity and elicits cellular and humoral immune responses	Preclinical stage	Septicemia	[454]
	4C-Staph/T7-alum	Protects against bacterial spread. It induces an increase in α-hemolysin neutralizing antibody levels and Th1 and Th17 profile responses. Induces the action of T CD4^+^ cells	Preclinical stage	Renal abscess and peritonitis	[455]
	Combination Al(OH)_3_, MPL, and WGP vaccine	Increases survival rate. Stimulates the innate immune system and induces epigenetic changes in macrophages that modulate phagocytosis and the inflammatory response to infection	Preclinical stage	Bacteremia and septicemia	[456]
	LukAB Toxoid	Directed against LukAB. Induces reduction of bacterial load at the surgical site and dissemination of *S. aureus*	Preclinical stage	Post-surgical infections	[457]
mAbs	Salvecin (AR-301—human anti-staphylococcal IgG mAb)	Directed against α-hemolysin	Clinical stage. Phase 1, 2, and 3 studies completed	Pneumonia (caused by MRSA and MSSA)	[458,459]
	Suvratoxumab (MEDI4893—human anti-α-toxin neutralizing mAb)	Directed against α-toxin, inhibiting its interaction with metalloprotease ADAM10 and its auto-oligomerization. Induces an increase in antibodies. In preclinical studies, it was responsible for increasing survival rates and reducing the bacterial load in the lungs	Clinical stage. Phase 1 and 2 studies completed, and phase 3 studies in progress.	Nosocomial pneumonia	([460,461,462,463]; ClinicalTrials.gov: NCT02296320, NCT05331885)
	DSTA4637S (anti-GlcNAc IgG mAb conjugated to a rifamycin class antibiotic)	Directed against the GlcNAc portion of teichoic acid from the cell wall of *S. aureus*. Targets intracellular *S. aureus*	Clinical stage. Phase 1 studies completed	*S. aureus* infections	([464,465]; ClinicalTrials.gov: NCT02596399)
	514G3	Directed against SpA. In preclinical studies it has been shown to stimulate IgG and IgA antibacterial responses and promote the decolonization of *S. aureus*	Clinical stage. Phase 2 studies completed	MRSA bacteremia	[466]
	Tefibazumab (Aurexis—humanized mAb that binds to the ClfA protein)	Directed against the adhesion protein ClfA expressed on the surface of *S. aureus*	Clinical stage. Phase 2 studies completed	Bacteremia and cystic fibrosis	([467]; ClinicalTrials.gov: NCT00198289)
	TRL1068	Targeted against highly conserved epitopes in the DNABII family of bacterial DNA-binding proteins (HU and IHF proteins).	Clinical stage. Phase 1 studies in progress	Joint infections	(ClinicalTrials.gov: NCT04763759)
	Linezolid (humanized anti-SEB neutralizing mAb)	Directed against SEB. Attenuates the systemic inflammatory response, protects against mortality, and inhibits the production of superantigens. Induces the production of antibodies	Preclinical stage	Pneumonia	[468]
	Dupilumab (humanized mAb anti-IL4Rα)	Directed against IL-4Rα. Significantly reduces bacterial load, serum CCL17, and disease severity. Reduces *S. aureus* cytotoxins and increases expression of genes relevant to Th17 responses, neutrophils, and complement pathways	Clinical stage. Phase 2 and 3 studies in progress	Atopic dermatitis	([469,470,471]; ClinicalTrials.gov: NCT03389893, NCT02277743, NCT02277769, NCT01859988)
Vaccines with mAb (epitope-focused immunization)	CgoX-D3 (anti-CgoX mAb)	Directed against the CgoX enzyme. It causes a strong and protective immune response against *S. aureus*. It has a wide effect	Preclinical stage	Septicemia	[472]
	TPI-H8 (anti-TPI mAb)	Directed against the TPI enzyme. It causes a strong and protective immune response against *S. aureus*. It has a wide effect	Preclinical stage	Septicemia	[472]
Other	Free-BR with MPS-NP	Directed against several virulence factors of *S. aureus*. It has antibacterial, anti-biofilm, anti-quorum sensing, and anti-virulence properties. Attenuates the expression of pro-inflammatory cytokines and pro-apoptotic genes	Preclinical stage	Multivirulent VRSA infections	[473]
	Genistein Isoflavone	Significantly suppresses bacterial growth and reduces the production of pro-inflammatory cytokines, such as TNF-α and IL-6	Preclinical stage	MRSA-induced osteomyelitis	[474]
	Butyrate	Attenuates skin inflammation aggravated by *S. aureus* by reducing IL-13 and leukocyte infiltration in the skin. Suppressed IL-33 expression and improved inflammation by inhibiting HDAC3	Preclinical stage	Atopic dermatitis	[475]
	Xuebijing	It improves survival and downregulates the expression of pro-inflammatory cytokines, such as IL-6, TNF-α, and IL-10. Reduces bacterial load and tissue damage. Downregulates the activation of NF-κB, MAPK, and PI3K/Akt in macrophages	Preclinical stage	Septicemia	[476]
	Sphingomyelin liposomes	Sequesters cytolytic toxins (phenol and α-hemolysin-soluble modulins) and prevents hemolysis and cell necrosis	Preclinical stage	MRSA Infections	[477]
	Angeli Salt	It inhibits mechanical hyperalgesia, edema, leukocyte migration, cytokine release, NF-κB activation, and oxidative stress. It reduces the severity of the disease and decreases the bacterial load	Preclinical stage	Septic arthritis	[118]
	Essential oils (trans-cinnamaldehyde, thymol, and carvacrol)	They have antimicrobial and antivirulence activities. They inhibit the growth of multivirulent *S. aureus* and MRSA. Downregulate the transcription of virulence genes	Preclinical stage	-	[478]

SA4Ag: *S. aureus* 4-antigen vaccine; CP5: capsular polysaccharide 5; CP8: capsular polysaccharide 8; rmClfA: recombinant surface protein clumping factor A; rMntC: recombinant manganese transport protein C; Als3: agglutinin-like sequence 3 surface protein; IL: interleukin; MRSA: methicillin-resistant *S. aureus*; SEB: staphylococcal enterotoxin B; MHC: major histocompatibility complex; LukS: S-subunit of Panton-Valentine Leucocidin; LukF: F-subunit of Panton-Valentine Leucocidin; LukAB: Leucocidin A/B; SEA: staphylococcal enterotoxin A; TSST-1: toxic shock syndrome toxin 1; GAS: ghosts of *S. aureus*; Al(OH)_3_: aluminum hydroxide; MPL: monophosphoryl lipid A; WGP: whole glucan particles; mAbs: monoclonal antibodies; MSSA: methicillin-sensitive *S. aureus*; ADAM10: disintegrin and metalloproteinase domain-containing protein 10; GlcNAc: N-acetyl-glucosamine; SpA: staphylococcal protein A; HU: histone-like proteins; IHF: integration host factor; IL-4Rα: interleukin-4 receptor; CCL17: Chemokine (C-C) ligand 17; CgoX: coproporphyrinogen III oxidase; TPI: triose phosphate isomerase; Free-BR: berberine free; MPS-NP: mesoporous silica nanoparticles; VRSA: vancomycin-resistant *S. aureus*; TNF-α: tumor necrosis factor-alpha; HDAC3: histone deacetylase 3; NF-κB: nuclear factor kappa B; MAPK: mitogen-activated protein kinase; PI3K/Akt: phosphoinositide 3-kinase/protein kinase B.
